# Striatal M_4_ muscarinic receptors determine the biological rhythm of activity, with a supportive role of M_1_ muscarinic receptors

**DOI:** 10.3389/fphar.2025.1691118

**Published:** 2025-12-01

**Authors:** Katerina Janisova, Monika Uhlirova, Sandor Forczek, Jaromir Myslivecek

**Affiliations:** 1 Institute of Physiology, 1^st^ Faculty of Medicine, Charles University, Prague, Czechia; 2 Isotope Laboratory, Institute of Experimental Botany, Academy of Sciences of the Czech Republic, Prague, Czechia

**Keywords:** M1 muscarinic receptors, M4 muscarinic receptors, cholinesterases, biological rhythm, intergeniculate leaflet, subparaventricular zone, suprachiasmatic nucleus

## Abstract

**Introduction:**

M_4_ muscarinic receptor (mAChR) knockout changed the female activity biological rhythm parameters. In this study, we focus on the biological rhythms of mAChRs (total + M_1_ mAChRs), acetylcholinesterase (AChE), and butyrylcholinesterase (BuChE) in M_4_ mAChR knockout (M_4_KO) and wild-type (WT) mice in specific brain areas.

**Methods:**

Female mice were sacrificed every 4 hours, brains were removed, mAChRs were determined by autoradiography, and punching was used for the measurement of acetylcholinesterase and butyrylcholinesterase activity. The density of mAChRs was correlated with locomotor activity.

**Results:**

An ultradian rhythm in total mAChRs was found in the suprachiasmatic nucleus (SCN) (both M_4_KO and WT). M_4_KO had a positive correlation between the number of mAChRs and locomotor activity. This rhythm was changed to circadian in WT with a peak in the active phase and to circadian rhythm in M_4_KO with phase shifts to the inactive/active phase in the intergeniculate leaflet (IgL) (positive correlation in KO), subparaventricular zone (SPVZ) (negative correlation in WT), and posterior hypothalamic area (PHA) (positive correlation in WT). The thalamus (TH) reveals circadian rhythms in WT and M_4_KO, with a peak in the active phase (no correlation). The striatum (Str), i.e., caudate ncl-putamen (CPu) (decrease in M_4_KO, positive correlation in both WT and KO) and the motor cortex (MCx) (no correlation), showed circadian rhythms (peak in active phase). Caudate ncl-putamen M_1_ mAChRs rhythm in WT was circadian, while M_4_KO animals revealed an ultradian rhythm. Cholinesterases revealed ultradian and circadian rhythms in different areas.

**Discussion:**

We conclude that muscarinic receptor-directed biological rhythm of activity is determined in the striatum (caudate ncl-putamen) as a key structure mainly by M_4_ mAChRs with a supportive role of M_1_ mAChRs.

## 1 Introduction

M_4_ muscarinic receptors (mAChRs) affect locomotor activity, and we showed (Valuskova et al., 2018b) that motor activity in M_4_KO female mice did not differ substantially from that of wild-type (WT) mice during the light period, but in the active (dark) phase, the M_4_KO mice were more active. They revealed an increase in the mesor, the night values, the night-day difference, and other biological rhythm parameters. As biological rhythm changes in the brain areas implicated in locomotor activity comprise more structures, we employed *in vitro* autoradiography and identified potential brain areas likely involved in biological rhythm regulation during locomotor activity (Valuskova et al., 2019) and important differences in morning vs. evening muscarinic drug (scopolamine and oxotremorine) effects, both in WT and M_4_ KO animals.

Furthermore [Riljak et al. (2020)], we searched for a potential mechanism of changes in the locomotor activity biological rhythm using constant darkness to distinguish light responsiveness from real circadian effects. We have shown that although core clock output is changed by M_4_ mAChRs deletion, the structures involved in biological rhythm regulation in WT and KO animals are likely the same (striatum (Str), thalamus (TH), and intergeniculate leaflet (IgL)). We also found that M_1_ mAChRs in the striatum (caudate ncl-putamen, CPu) are implicated in the regulation of the locomotor activity biological rhythm.

The description of projections and connections involved in the regulation of locomotor activity is complicated due to the many mutual interconnections. For details, please see Table 1 and the relevant reviews and original articles: Watts and Swanson (1987); Watts et al. (1987); Moga et al. (1995); Edelstein and Amir (1996); Moore (1996); Krout et al. (2002); Abrahamson and Moore (2006); Morin and Allen (2006); Morin (2013); and Vujovic et al. (2015). Other connections (thalamostriatal, striatocortical, and thalamocortical) are well-known and constitute textbook knowledge.

**TABLE 1:** Projections and connections between brain areas involved in the regulation of the locomotor activity. The table is limited to the main pathways. Other connections (striatum–thalamus, thalamus–cortex, and cortex–striatum) are not shown. A simplified scheme of these interconnections is included in summarizing Figure 12.

Output	Input	Note	References
Retina	SCN	Direct	Edelstein and Amir (1996)
Retina	IgL	Collateral	Morin and Allen (2006)
Retina	SPVZ	Collateral	Morin (2013)
SCN	SPVZ	Dense projections	Watts and Swanson (1987)
IgL	SCN	Photic input *Geniculohypothalamic tract*	Edelstein and Amir (1996)
IgL	SCN SPVZ anterior hypothalamic area Pretectal area Paraventricular thalamus 1		Moore (1996)
IgL	Lateral hypothalamus Posterior hypothalamus Anterodorsal thalamic nuclei Centromedian thalamic nuclei Centrolateral thalamic nuclei Anterior paraventricular thalamic nuclei Olfactory tubercle Lateral olfactory tract nuclei		Morin (2013)
SCN	PHA		Watts et al. (1987)
SCN	Thalamus	e.g., Paraventricular nucleus	Moga et al. (1995)
SPVZ	SCN		Krout et al. (2002)
SPVZ	Thalamus		Vujovic et al. (2015)
Thalamus	SCN		Moga et al. (1995)
PHA	Cortex		Abrahamson and Moore (2006)

The cholinergic innervation of the suprachiasmatic nucleus (SCN) arises from the cholinergic forebrain and brain stem nuclei. The density of cholinergic fibers and terminals is modest compared to other hypothalamic nuclei (Hut and Van der Zee, 2011). As demonstrated earlier by Valuskova et al. (2018a) and Riljak et al. (2020), M_4_ mAChRs are abundant in the striatum (caudate ncl-putamen, 46% of all mAChRs). There was also a relatively high density of M_1_ mAChRs (37%). Similarly, a high percentage of M_4_ mAChRs (43%) is present in the thalamus. However, the total muscarinic population in the thalamus is low.

The cholinergic system manifests rhythmical activity. However, the data obtained are not identical; some differences originate from different structures or species studied (see Liu and Gillette (1996); Bina et al. (1998); Buchanan and Gillette (2005)).

Cholinergic enzymes (choline acetyltransferase and acetylcholinesterase (AChE)) measured *postmortem* in the human brain (Perry et al., 1977) showed the amplitude and phases of the changes differently. Circadian fluctuations of cholinergic enzyme activity were reviewed (Hut and Van der Zee, 2011). They found that in most brain areas, during the active phase, there is a peak of choline acetyltransferase activity, while AChE activity has its peak in the inactive phase.

The findings on mAChRs reveal differences among studies. The levels of mAChRs (in the cortex) measured *postmortem* in the human brain (Perry et al., 1977) were higher in the inactive phase. In the review by Hut and Van der Zee (2011), most animal (rodent) brain areas were reported to have mAChRs peaking in the inactive phase. In other studies or reviews on rats (Wirz-Justice et al., 1981; Kafka et al., 1983; Wirz-Justice, 1987), the peak of mAChRs (forebrain) circadian rhythm was reported to occur during the active period. Circadian rhythmicity of mAChRs was detected in the rat hippocampus and the hypothalamus (not in the cortex, striatum, or cerebellum (Por and Bondy, 1981)). Biological rhythms in mAChRs (with a peak in the active phase) were found in the rat parietal cortex and caudate-putamen (Kafka et al., 1986), and no rhythms were found in other areas. In another study (Marquez et al., 1990), the peak of mAChRs was in the non-active period of rats (at 14:00 h).

Different brain areas show distinct reactions to muscarinic agonists/antagonists. However, one should take into account that the binding of orthosteric agonists/antagonists to different mAChR subtypes is similar (Myslivecek, 2022). For details concerning this issue, see the Discussion.

In this study, we aimed to determine the key structure for the biological rhythms of locomotor activity by studying the rhythms of mAChRs (the total number of mAChRs and M_1_ mAChRs) and the activity of cholinesterases (AChE and butyrylcholinesterase (BuChE)) in brain areas previously identified as structures involved in the regulation of the biological rhythm during motor activity: IgL, striatum (caudate ncl-putamen), and thalamus. In addition to that, we determined the cholinergic markers in the following structures: SCN, subparaventricular zone (SPVZ), posterior hypothalamic area (PHA), and motor cortex (MCx). We also aimed to reveal the sequence of changes in these brain areas.

## 2 Methods

### 2.1 Animals

Mice lacking the M_4_ muscarinic receptor were generated in Wess’s laboratory (Gomeza et al., 1999) and were subsequently bred in our animal facility (Prague, Czech Republic). Their genetic background was C57Bl/6NTac (WT animals). The animals were treated in accordance with the legislature of the Czech Republic and the EU legislature (European Convention for the Protection of Vertebrate Animals Used for Experimental and Other Scientific Purposes [Council of Europe No 123, Strasbourg 1985]), and the experimental protocol was approved by the Committee for the Protection of Experimental Animals of the First Medical Faculty, Charles University, Prague, and the Ministry of Education of the Czech Republic under No MSMT-2409/2017-3 and MSMT-5939/2022-4. We studied fully backcrossed (15 generations) M_4_−/− and M_4_ +/+ mAChRs littermates. The animals were maintained under controlled environmental conditions (12/12-h light/dark cycle, 22 °C ± 1 °C, light on at 6:00). Food and water were available *ad libitum*. A total of 144 female mice (weighing 20–26 g, age 3–6 months) were used in the study, of which there were 72 M_4_ KO animals and 72 WT animals. Before all the experiments, the mice were carefully genotyped, and only homozygous mice were used. The control is carried out by genotyping the tissue taken when the animal is sacrificed at the end of the experiment. The female mice were housed separately from male mice, thus revealing the Lee–Boot effect (i.e., suppression of the estrus cycle—anestrous), which made the female group homogeneous in hormone levels. Moreover, no differences were observed in light microscopy of vaginal lavage or actograms in female mice for 15 consecutive days (control animals were not included in the experiment because of the stressful procedure associated with lavage acquisition).

### 2.2 Tissue preparation

The tissue of several brain areas previously shown to relate to locomotor activity biological rhythm changes (MCx, CPu, TH, SCN, SPVZ, PHA, and IgL) was used in this experiment. As the striatum consists of two distinctively different structures, we also compared the muscarinic receptor density in caudate ncl-putamen with the density in the ncl. accumbens. The mice were euthanized using cervical dislocation and decapitation, with the aim of not harming receptors using anesthesia. Brains were rapidly removed—12 brains per group every 4 hours (i.e., at 2:00, 6:00, 10:00, 14:00, 18:00, and 22:00), frozen in dry ice, and then stored at −80 °C. Individual structures (SCN, SPVZ, IGL, MCx, CPu, TH, and PHA) were verified in Nissl staining (see Supplementary Figure S1). For details, see Valuskova et al. (2018b). In brief, the parallel sections were obtained using a cryostat, and the area, clearly visible as in Nissl staining, was then marked (using border transposition) on a scanned autoradiogram (see section 2.5) and used for densitometry with PC-based analytical software.

### 2.3 Total muscarinic receptor number

Muscarinic receptors were detected using the non-specific antagonist ^3^H-QNB (^3^H-quinuclidinyl benzilate or ^3^H-1-azabicyclo [2.2.2]oct-3-yl 2-hydroxy-2,2-diphenylacetate, 50.5 Ci/mmol, PerkinElmer, Inc., United States) in a radioligand binding assay. Autoradiography was performed as described previously in detail (Farar et al., 2012); the sections were incubated for 90 min with 2 nM ^3^H-QNB at room temperature. As a control, we performed competitive incubation of ^3^H-QNB (170 pmol/L, value within range 1.0-2.0 fold estimated K_D_) in the CPu of M_4_ KO animals with selective M_4_ antagonist VU6013720 (synthesized by Santiago, Inc., Czech Republic; final concentration 30 nmol/L, the concentration blocking M_4_ mAChRs but not the other mAChRs) (Moehle et al., 2021; Qi et al., 2023). Moreover, as another control, we performed saturation binding in the motor cortex with ^3^H-QNB (0.0625-2 nmol/L), pre-incubated with 30 nmol/L VU6013720.

### 2.4 M_1_ mAChR number

For M_1_ mAChR receptor number determination, the specific protocol of autoradiography was performed (Valuskova et al., 2018a), and the sections were incubated for 60 min with 5 nM ^3^H-pirenzepine (non-specific antagonist, ^3^H-11-[(4-methylpiperazin-1-yl)acetyl]-5,11-dihydro-6H-pyrido[2,3-b][1,4]benzodiazepin-6-one, 100.0 Ci/mmol; American Radiolabeled Chemicals, Inc., United States) at room temperature (RT).

### 2.5 Common procedure for autoradiography

Frontal sections (16-micrometer-thick) were cut on a cryostat at −20 °C, thaw-mounted onto Superfrost® Plus glass slides (Carl Roth GmbH and Co. KG, Karlsruhe, Germany), and stored in boxes at − 80 °C until use. To assess mAChR binding (total and M_1_ mAChRs), the sections were allowed to thaw and dry for 30 min at 22 °C, and the density of receptors was determined as follows. Dry brain sections were pre-incubated for 30 min in 50 mM potassium phosphate buffer (pH 7.4) at RT. Incubation with specific ligands is mentioned above. Nonspecific binding was assessed on adjacent sections in the presence of 10 μM atropine sulfate. After incubation, the sections were washed twice for 5 min and twice for 2 seconds and then gently dried. Dry sections were apposed to the tritium-sensitive Fuji BAS imaging plates (GE Healthcare Europe GmbH, Freiburg, Germany) in Kodak Biomax autoradiographic cassettes (Carestream Health, Inc., Rochester, NY, United States) for 5 days. The linearity of the signal and conversion of photo-stimulated luminescence to radioactivity were assessed using tritium autoradiographic standards (American Radiolabeled Chemicals, Inc., St. Louis, MO, United States). The film autoradiograms were scanned, and densitometry was performed with the PC-based analytical software, MCID analysis software. Measurements were taken from at least three sections for each animal and brain region, and the values were then averaged. We compared the densities in the left and right hemispheres. Since there were no differences in laterality, both sides were taken together.

### 2.6 Acetylcholinesterase and butyrylcholinesterase activity

The tissue for enzyme activity analysis was obtained every 4 h (i.e., at 2:00, 6:00, 10:00, 14:00, 18:00, and 22:00) using a micro-punching technique similar to that used earlier (Valuskova et al., 2017). In detail, samples of the MCx, Str, and TH were isolated using a punch method, collecting as much tissue as possible. Brains were quickly removed, immediately frozen on dry ice, and kept in storage boxes at −80 °C until needed. The brains were cut into 3-mm-thick serial coronal sections with a razor blade with a handle (P-lab a. s., Prague, Czech Republic) using a cryostat chamber at a temperature of −10 °C. The tissue was isolated using specialized metal punching needles and then stored at −80 °C until further analysis.

Tissue preparation: Isolated brain tissue was homogenized using an Ultra Turrax Homogenizer (IKA-Werke GmbH & Co. KG, Staufen, Germany) by three pulses of 10 s in 300 μL of 0.32 M sucrose at 4 °C. The activity of AChE and BuChE was determined by Ellman’s colorimetric method, modified for a 96-well microtiter plate reader (Tecan Sunrise, Tecan Group Ltd, Männedorf, Switzerland), as previously described (Valuskova et al., 2017). The activity of AChE was assayed with 0.69 mM acetylthiocholine and 0.5 mM 5,5-dithiobis (2-nitrobenzoic acid) (DTNB) in 5 mM HEPES buffer, pH 7.4, containing 10 mM MgCl_2_. Tissue samples (10 μg) were first pre-incubated with DTNB to saturate free sulfhydryl groups and subsequently with tetra (monoisopropyl)pyrophosphortetramide (iso-OMPA) (final concentration 0.1 mM) to block AChE activity for 30 min. The activity was measured at 412 nm at different time points (0, 5, 10, 20, and 30 min). BuChE activity was assayed as described for AChE except that butyrylthiocholine was used as a substrate and BuChE activity was blocked with 1,5-bis(4-allyldimethylammoniumphenyl)pentan-3-one dibromide (BW284C51) (final concentration 5 μM). The total assay volume was 200 μL. Protein concentration was determined using a BCA assay kit (Pierce, Waltham, MA). The amount of protein was identical for all brain regions (μg prot./ml).

The amount of reaction product was calculated according to the Beer–Lambert law, as shown in Equation 1.

A=εbc, (1)

where A is absorbance, b is the path length in centimeters (cm), c is the concentration in moles/liter (M), and ε is the molar extinction coefficient in (M^-1^cm^-1^).

### 2.7 Biological rhythm calculations and statistical evaluation

For the biological rhythm calculations, the nature of the obtained data (autoradiography performed at specific time points–every 4 h) does not allow the use of Cosinor analysis. This could bias the results. Therefore, we only labeled the peaks and described the rhythm according to whether it was circadian.

The Cosinor analysis, which we performed for control, did indeed bias the resulting data (fitting multiple peaks, giving different values, or software marked the data as insufficient to conduct a valid analysis).

Furthermore, as a control, we used the advanced mathematical functions of MATLAB R2022b to obtain 24 points for chronological evaluation. MATLAB accomplished this using cubic spline interpolation (polynomial of degree 3). We chose to use this special case of spline interpolation because this interpolation polynomial is smoother, has less error than some other polynomials, and also helps avoid the Runge effect (using a higher degree polynomial does not always improve accuracy; instead, oscillations appear at the edges of the interval). Thus, our presented results only appear to be simply sampling the time point with the largest value. The interpreted results are based on verification using this interpolation method.

Individual values were calculated from the respective phases of the day (day and night). The specific biological rhythm values were calculated: day mean (D_mean_), night mean (N_mean_), the difference between night and day mean (N-D_mean_), and mesor (midline value). These values, as they depend on each other, were compared in WT and M_4_KO animals using two-way ANOVA in GraphPad Prism software with Sidak’s post-hoc correction. Statistically, it is necessary to analyze the parameters as a whole to avoid bias (false statistical significance) between groups when performing a significance analysis between two groups. Since the samples were obtained post-mortem, comparisons between individual time points cannot be applied, only comparisons between groups.

To determine which structure is important for locomotor activity biological rhythm, we have correlated locomotor activity data (see the original locomotor activity graph in Figure 11) obtained earlier (Valuskova et al., 2018b) with present data on mAChR densities in specific structures. Linear regressions were also calculated.

### 2.8 Relative m4 mAcChR mAChR density

The changes in the relative density of M4 mAChRs were counted accodring to Equation 2 as the difference between the total mAChR density in WT animals and M4 KO animals. The difference between the total number of binding sites in WT and the total number of binding sites in KO animals determines the relative density of M4 mAChRs:

$$M_{4}\text{ mAChRs density}=\text{mAChRs }{\text{density}}_{\text{WT}}-\text{mAChRs }{\text{density}}_{\text{KO}}$$ (2)

## 3 Results

The incubation of ^3^H-QNB with VU6013720 (M_4_ mAChRs specific antagonist) in CPu showed no reduction in the binding in M_4_KO animals: 755.5 ± 479.5 fmol/mg protein (M_4_KO) vs. 733.3 ± 272.2 fmol/mg protein (M_4_KO + VU6013720), while in WT animals, VU6013720 caused a reduction in binding from 2000 ± 183.7 fmol/mg protein to 748.1 ± 297.4 fmol/mg protein (by 63%). Saturation binding in the motor cortex with ^3^H-QNB pre-incubated with VU6013720 showed a reduction of binding in WT animals by 34.26% (1,412 ± 341.5 fmol/mg prot. vs. 928.2 ± 121.8 fmol/mg. prot., respectively), while in KO, there was no reduction in binding (802 ± 160.6 fmol/mg prot. vs. 772.8 ± 115.9 fmol/mg prot.).

### 3.1 Total number of mAChRs

The representative autoradiograms in WT and M_4_KO animals are shown in Supplementary Figure S2.

#### 3.1.1 Suprachiasmatic nucleus

**FIGURE 1:**
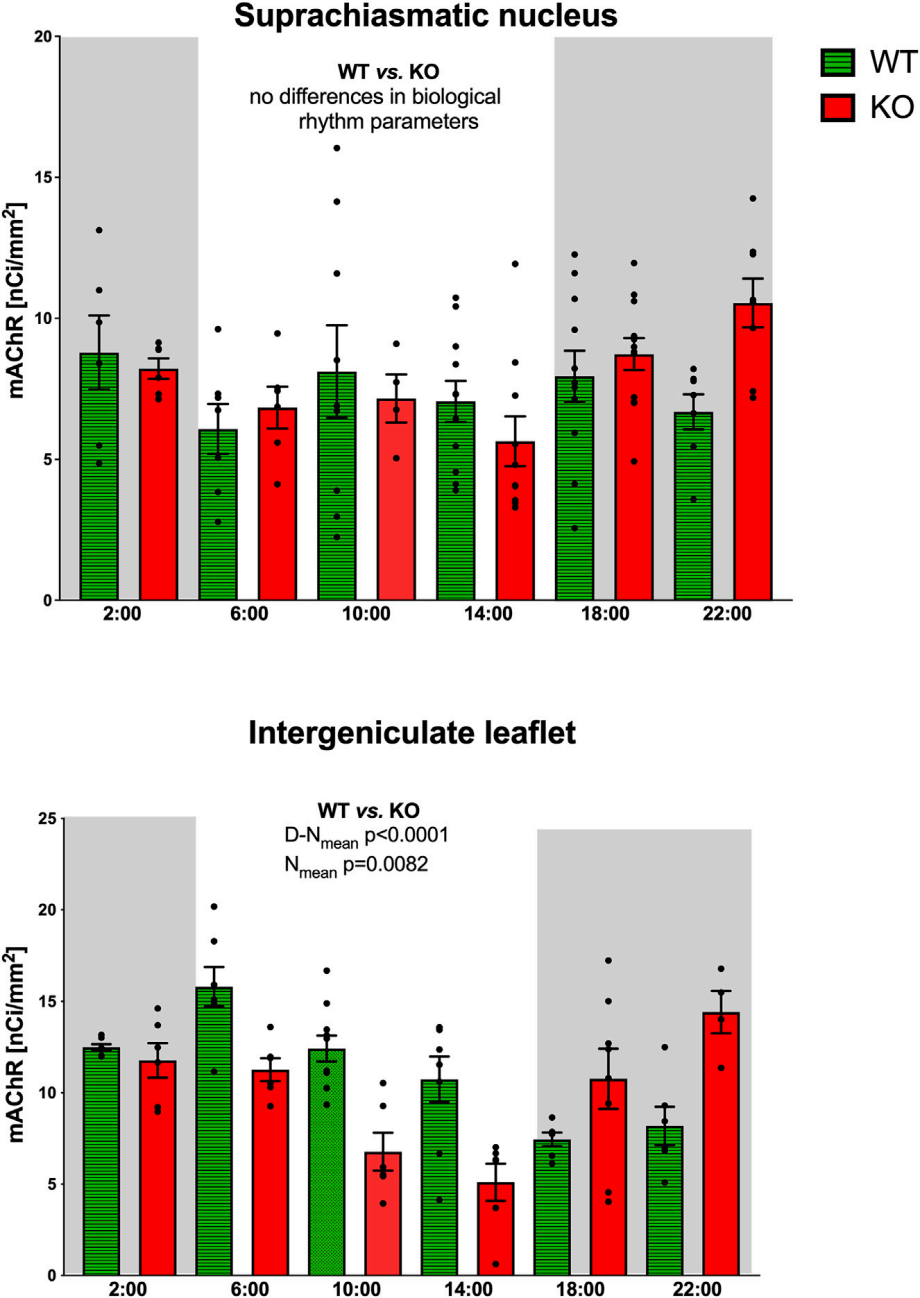
Top: the changes in the total number of mAChRs in the suprachiasmatic nucleus. Ordinate: relative density expressed as nCi/mm^2^; abscissa: time [h]. Grey rectangles represent the dark phase (lights off at 18:00 and lights on at 6:00). Data are expressed as the mean ± SEM with individual points. Two-way ANOVA: interaction between the genotype and biological rhythm parameters was not significant: F_3,140_ = 0.72, *p* = 0.54. The result of statistical analysis is given in the middle of the figure. Bottom: the changes in the total number of mAChRs in the intergeniculate leaflet. Ordinate: relative density expressed as nCi/mm^2^; abscissa: Abscissa: time [h]. Grey rectangles represent the dark phase (lights off at 18:00, lights on at 6:00). Data are expressed as the mean ± SEM with individual points. Two-way ANOVA: interaction between the genotype and biological rhythm parameters was significant: F_3,144_ = 12.10, *p* < 0.0001, and differences have been shown in N_mean_ (*p* = 0.008) and N–D_mean_ (*p* < 0.0001). The result of statistical analysis is given in the middle of the figure. Legend: WT, wild-type animals, KO, M_4_ mAChR KO animals.

**FIGURE 2:**
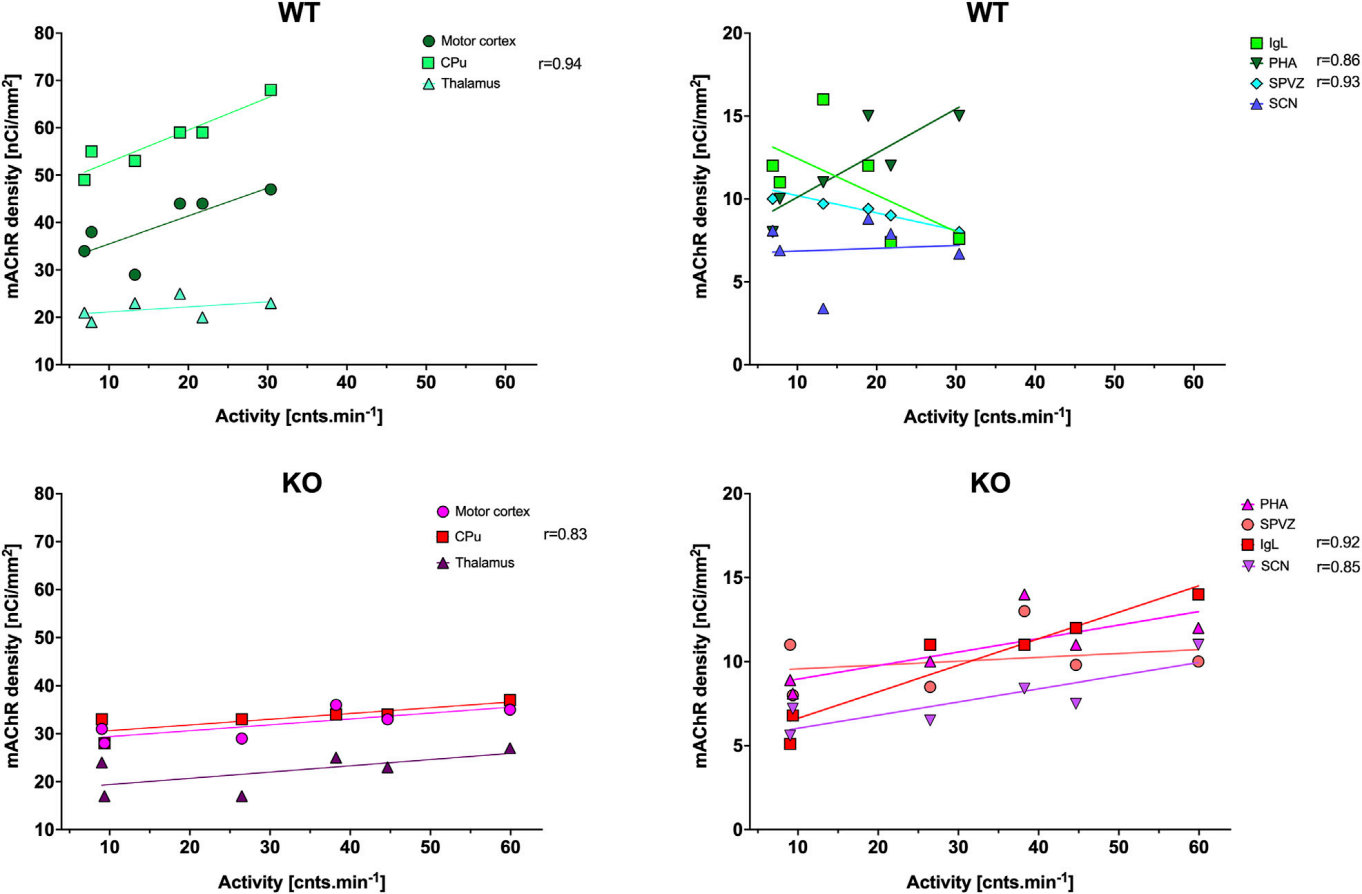
Correlation between the total number of mAChRs and locomotor activity (as published previously by Valuskova et al. (2018b), which is shown in Figure 11). Top: the linear regression in WT animals in the motor cortex, CPu, and thalamus (left) and in IgL, PHA, SPVZ, and SCN (right, see list of abbreviations). Bottom: the linear regression in KO animals in the motor cortex, striatum, and thalamus (left) and in IgL, PHA, SPVZ, and SCN (right). Ordinate: relative density expressed as nCi/mm^2^; abscissa: activity [cnts.min^-1^]. Please note that in KO animals, there was higher locomotor activity. Significant correlation coefficients are shown.

In the SCN, the biological rhythm of mAChR total number revealed ultradian variations in both WT and M_4_KO animals (Figure 1). The peaks in WT animals were at 2:00, 10:00, and 18:00, while in M_4_KO animals, there were peaks at 10:00 and 22:00. There was no correlation between locomotor activity and the total number of mAChRs in WT (Pearson r = 0.08, *p* = 0.88), while in KO animals, there was a strong correlation (r = 0.85, *p* = 0.03). Linear regression (see Figure 2) showed a slope of 0.02, not significantly different from zero (F_DFn_, _DFd_ was (F_1,4_ = 0.0258, *p* = 0.8799, where DFn where DFn is the degrees of freedom numerator and DFd is the degrees of freedom denominator) in WT. In KO, the slope was 0.08, which was significantly different from zero (F_1,4_ = 10.06, *p* = 0.034).

Statistical analysis of biological rhythm parameters (two-way ANOVA: interaction of two factors (genotype (WT vs. KO) and biological rhythm parameters (N-D_mean_, N_mean_, N–D_mean_, and Mesor) was not significant: F_3,140_ = 0.72, *p* = 0.54. Thus, Sidak’s post-hoc test was not appropriate (see also Table 2).

**TABLE 2:** Biological rhythm parameters of mAChR total number in specific brain areas.

	WT				KO			
	D_mean_	N_mean_	N–D_mean_	Mesor	D_mean_	N_mean_	N–D_mean_	Mesor
SCN	5.5 ± 0.66 (n = 21)	6.7 ± 0.37 (n = 21)	1.3 ± 0.49 (n = 21)	6.1 ± 0.48 (n = 21)	6.2 ± 0.62 (n = 16)	7.8 ± 0.41 (n = 16)	1.6 ± 0.5 (n = 16)	6.8 ± 0.5 (n = 16)
IgL	14 ± 1.1 (n = 20)	9.3 ± 0.82 (n = 20)	−4.3 ± 0.7 (n = 20)	12.0 ± 0.91 (n = 20)	11.0 ± 1.2 (n = 18)	14.0 ± 1.4 (n = 18)**	3.0 ± 0.43 (n = 18) ****	12.0 ± 1.3 (n = 18)
PHA	11 ± 0.98 (n = 24)	12.0 ± 1.1 (n = 24)	0.85 ± 0.98 (n = 24)	11.0 ± 0.75 (n = 24)	8.5 ± 0.7 (n = 26)	9.7 ± 0.46 (n = 26)	1.2 ± 0.77 (n = 26)	9.2 ± 0.46 (n = 26)
SPVZ	10 ± 0.65 (n = 14)	7.5 ± 0.43 (n = 14)	−2.6 ± 0.43 (n = 14)	8.8 ± 0.5 (n = 14)	7.6 ± 0.58 (n = 15)**	10.0 ± 0.57 (n = 15) **	2.7 ± 0.22 (n = 15) ****	9.0 ± 0.56 (n = 15)
Thalamus	18.0 ± 1.3 (n = 26)	21.0 ± 1.3 (n = 26)	3.5 ± 0.82 (n = 26)	19.0 ± 1.3 (n = 26)	18.0 ± 1.2 (n = 21)	22.0 ± 1.9 (n = 21)	4.8 ± 1.1 (n = 21)	20.0 ± 1.5 (n = 21)
Striatum	54.0 ± 4.2 (n = 36)	63.0 ± 5.3 (n = 36)	8.3 ± 1.6 (n = 36)	59.0 ± 4.7 (n = 36)	31.0 ± 1.3 (n = 32) ****	33.0 ± 2.1 (n = 32) ****	1.8 ± 1.1 (n = 32)	32.0 ± 1.6 (n = 32) ****
Motor cx.	35.0 ± 3.1 (n = 35)	42.0 ± 3.3 (n = 35)	6.3 ± 0.99 (n = 35)	38.0 ± 3.2 (n = 35)	27.0 ± 2.1 (n = 28)	35.0 ± 2.0 (n = 28)	7.1 ± 0.61 (n = 28)	31.0 ± 2.0 (n = 28)

Abbreviation—see text. Difference from WT: **p < 0.01; ****p < 0.0001.

#### 3.1.2 Intergeniculate leaflet

The ultradian variations visible in the SCN were changed to a circadian rhythm in the IgL in both WT and KO animals (Figure 1). However, the M_4_KO animals had a peak value shifted (advanced) by 8 h. While the WT animals had a peak value at 6:00 (end of the active phase), M_4_KO had a peak at 22:00 (middle of the active phase). Two-way ANOVA (interaction) (F_3,144_ = 12.10, *p* < 0.0001) showed differences in N_mean_ (*p* = 0.008) and N–D_mean_ (*p* < 0.0001) (see also Table 2). There was no correlation between activity and the total number of mAChRs in WT (r = −0.62, *p* = 0.19), while in KO animals, there was a strong correlation (r = 0.92, *p* = 0.004). Linear regression (see Figure 2) showed a slope of −0.22 in WT, which was not significantly different from zero (F_1,4_ = 2.495, *p* = 0.19). In KO, the slope was 0.16, which was significantly different from zero (F_1,4_ = 37.86, *p* = 0.004).

#### 3.1.3 Posterior hypothalamic area

**FIGURE 3:**
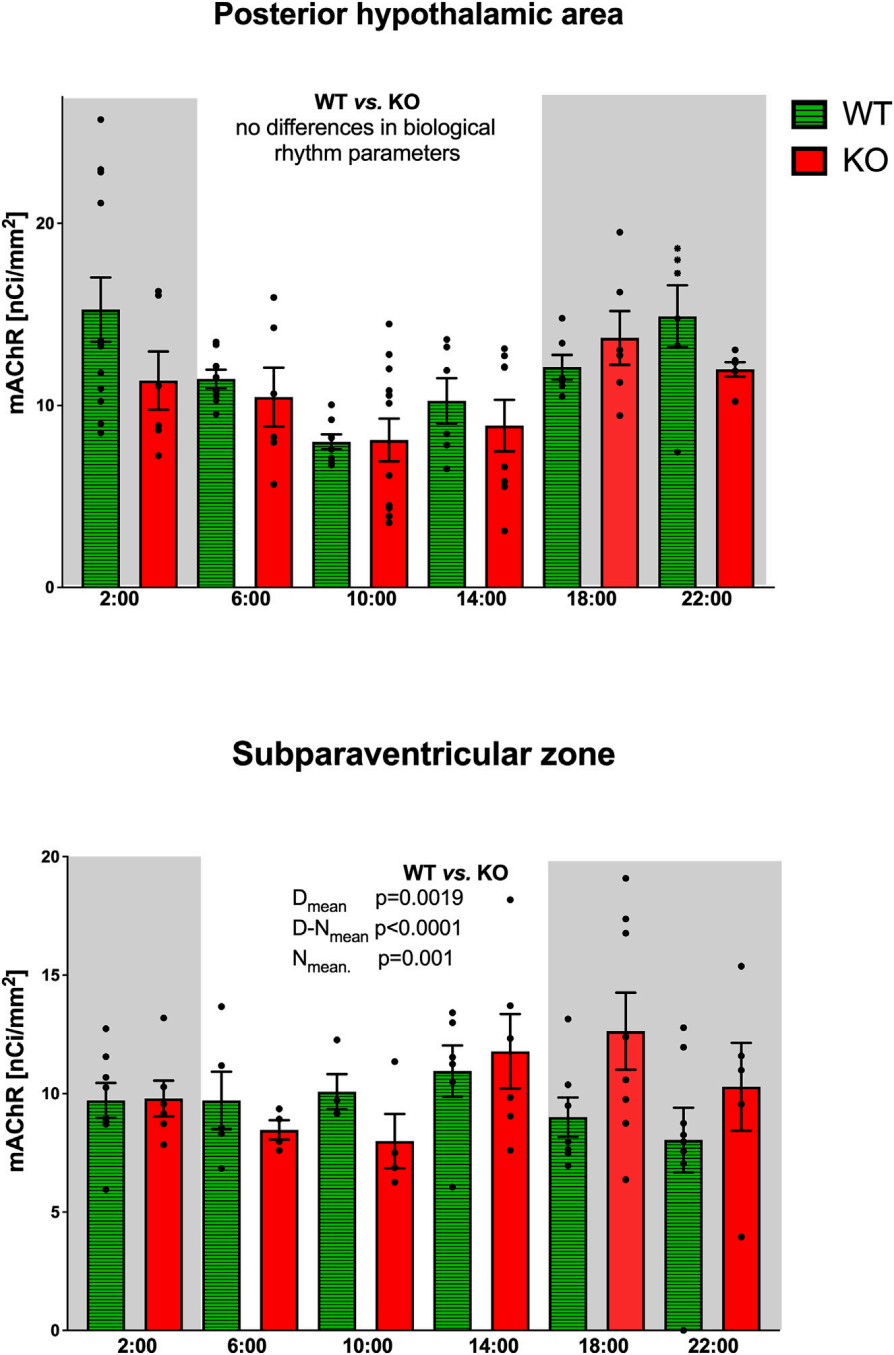
Top: the changes in the total number of mAChRs in the posterior hypothalamic area. Ordinate: relative density expressed as nCi/mm^2^; abscissa: time [h]. Grey rectangles represent the dark phase (lights off at 18:00 and lights on at 6:00). Data are expressed as the mean ± SEM with individual points. Two-way ANOVA: interaction between the genotype and biological rhythm parameters was not significant: F_3,192_ = 0.8865, *p* = 0.45. The result of statistical analysis is given in the middle of the figure. Bottom: the changes in the total number of mAChRs in the subparaventricular zone. Ordinate: relative density expressed as nCi/mm^2^; abscissa: time [h]. Grey rectangles represent the dark phase (lights off at 18:00 and lights on at 6:00). Data are expressed as the mean ± SEM with individual points. Two-way ANOVA: interaction between the genotype and biological rhythm parameters was significant: F_3,108_ = 23.26, *p* < 0.0001 and differences have been shown in the N_mean_ (*p* = 0.001), D_mean_ (*p* = 0.0019), and N–D_mean_ (*p* < 0.0001). The result of statistical analysis is given in the middle of the figure. Legend: WT, wild-type animals; KO, M_4_ mAChR KO animals.

As with the IgL, the PHA also revealed circadian variations in both WT and KO animals (Figure 3). The M_4_KO animals had a peak value shifted (advanced) by 8 h. While the WT animals had a peak value at 2:00 (middle of the active phase), M_4_KO had a peak at 18:00 (beginning of the active phase). Two-way ANOVA (interaction) (F_3,192_ = 0.8865, *p* = 0.45) showed no difference between WT and M_4_KO animals (see also Table 2). There was a correlation between locomotor activity and the total number of mAChRs in WT (r = 0.86, *p* = 0.028), while in KO animals, there was no correlation (r = 0.76, *p* = 0.08). Linear regression (see Figure 2) showed a slope of 0.27 in WT, which was significantly different from zero (F_1,4_ = 11.51, *p* = 0.028). In KO, the slope was 0.08, which was not significantly different from zero (F_1,4_ = 5.34, *p* = 0.082).

#### 3.1.4 Subparaventricular zone

The subparaventricular zone also revealed a circadian rhythm in the total number of mAChRs (Figure 3). The M_4_KO animals had a peak value shifted (delayed) by 4 h. While the WT animals had a peak value at 14:00 (in the inactive phase), M_4_KO had a peak at 18:00 (beginning of the active phase). Two-way ANOVA (interaction) (F_3,108_ = 23.26, *p* < 0.0001) showed differences between D_mean_ (*p* = 0.0019), N_mean_ (*p* = 0.001), and N–D_mean_ (*p* < 0.0001) (see also Table 2). There was a correlation between locomotor activity and the total number of mAChRs in WT (r = −0.93, *p* = 0.007), while in KO animals, there was no correlation (r = 0.26, *p* = 0.62). Linear regression (see Figure 2) showed a slope of −0.104 in WT, significantly different from zero (F_1,4_ = 27.14, *p* = 0.007). In KO, the slope was 0.02, which was not significantly different from zero (F_1,4_ = 0.28, *p* = 0.62).

#### 3.1.5 Thalamus

**FIGURE 4:**
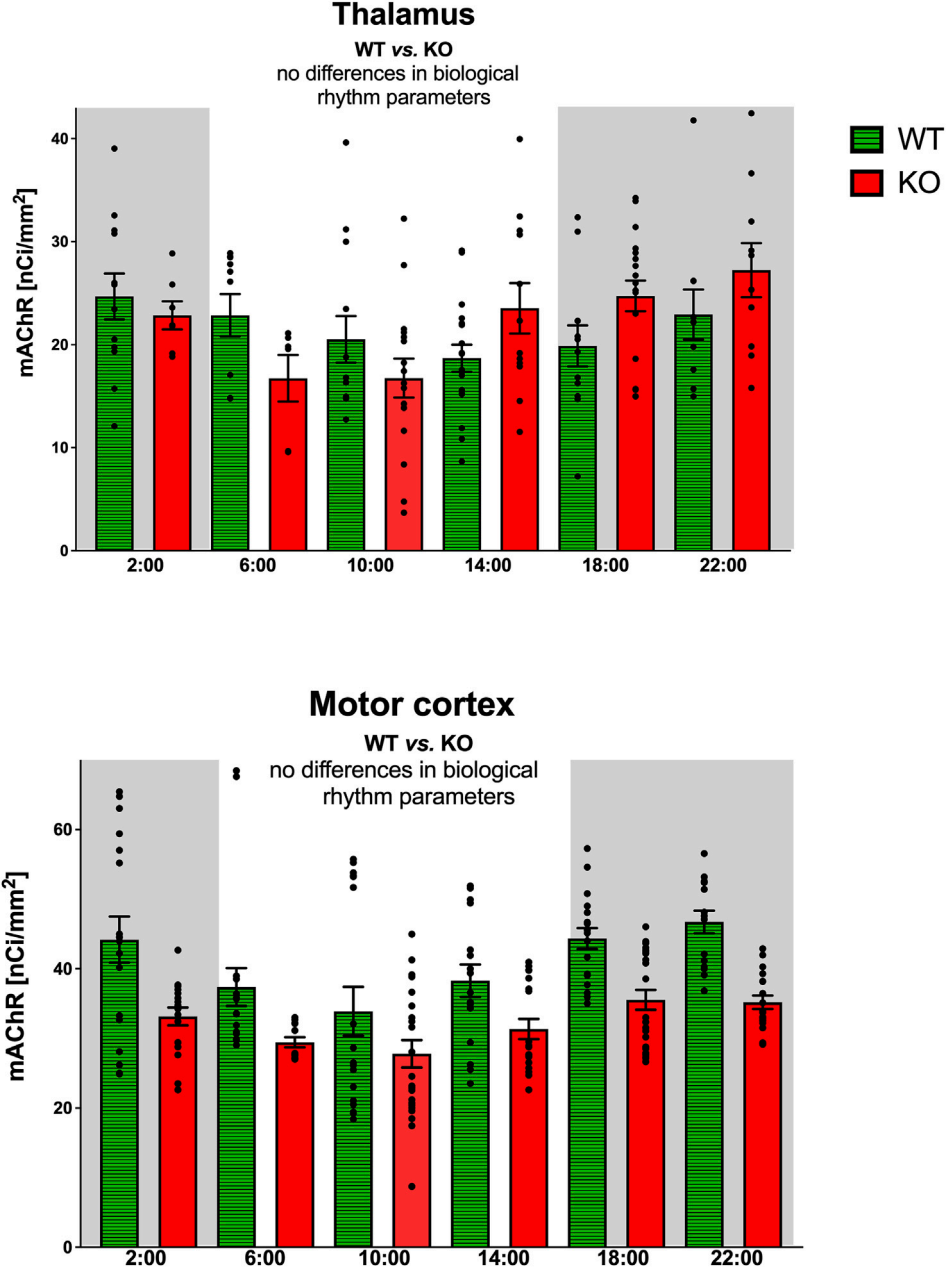
Top: the changes in the total number of mAChRs in the thalamus. Ordinate: relative density expressed as nCi/mm^2^; abscissa: time [h]. Grey rectangles represent the dark phase (lights off at 18:00 and lights on at 6:00). Two-way ANOVA: interaction between the genotype and biological rhythm parameters was not significant: F_3,180_ = 0.36, *p* = 0.78. Data are expressed as the mean ± SEM with individual points. See legend for an explanation of symbols. Bottom: the changes in the total number of mAChRs in the motor cortex. Ordinate: relative density expressed as nCi/mm^2^; abscissa: time [h]. Grey rectangles represent the dark phase (lights off at 18:00 and lights on at 6:00). Data are expressed as the mean ± SEM with individual points. Two-way ANOVA: interaction between the genotype and biological rhythm parameters was not significant: F_3,244_ = 0.85, *p* = 0.47. The result of statistical analysis is given in the middle of the figure. Legend: WT, wild-type animals; KO, M_4_ mAChR KO animals.

The thalamus was also the brain structure with circadian variations in the total number of mAChRs (Figure 4). The M_4_KO animals had a peak value shifted (advanced) by 4 h. While the WT animals had a peak value at 2:00 (middle of the active phase), M_4_KO had a peak at 22:00 (in the active phase). Two-way ANOVA (interaction) (F_3,180_ = 0.3584, *p* = 0.78) did not find any differences between WT and M_4_KO animals (see also Table 2). There was no correlation between activity and the total number of mAChRs in WT (r = 0.43, *p* = 0.39). Similarly, in KO animals, there was also no correlation (r = 0.63, *p* = 0.18). Linear regression (see Figure 2) showed a slope of 0.11 in WT, which was not significantly different from zero (F_1,4_ = 0.91, *p* = 0.39). In KO, the slope was 0.13, which was not significantly different from zero (F_1,4_ = 2.57, *p* = 0.18).

#### 3.1.6 Caudate ncl-putamen

**FIGURE 5:**
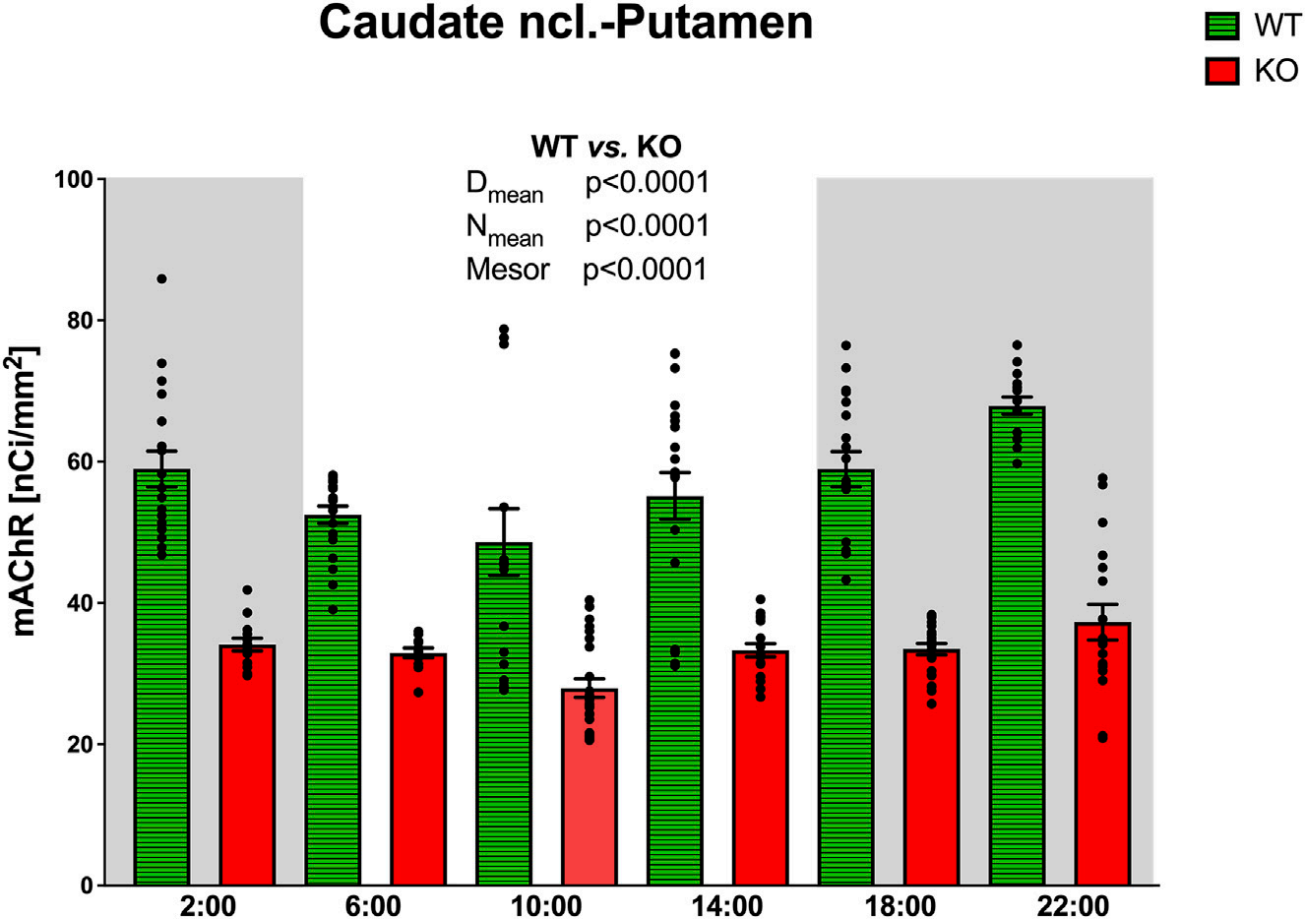
Changes in the total number of mAChRs in the CPu. Ordinate: relative density expressed as nCi/mm^2^, abscissa: time [h]. Grey rectangles represent the dark phase (lights off at 18:00 and lights on at 6:00). Data are expressed as the mean ± SEM with individual points. Two-way ANOVA: interaction between the genotype and biological rhythm parameters was significant: F_3,264_ = 12.57, *p* < 0.0001, and differences have been shown in the N_mean_ (*p* < 0.0001), D_mean_ (*p* < 0.0001), and mesor (*p* < 0.0001). The result of statistical analysis is given in the middle of the figure. Legend: WT, wild-type animals; KO, M_4_ mAChR KO animals.

CPu revealed circadian rhythmicity in total mAChRs in both WT and M_4_KO animals, with a significant decrease in mAChRs in M_4_KO animals (Figure 5). Both rhythms had a peak in the active phase at 22:00. Two-way ANOVA (interaction) (F_3,264_ = 12.57, *p* < 0.0001) showed differences between D_mean_ (*p* < 0.0001), N_mean_ (*p* < 0.0001), and mesor (*p* < 0.0001) (see also Table 2). There was a strong correlation between locomotor activity and the total number of mAChRs in WT (r = 0.94, *p* = 0.006), and similarly, in KO animals, there was also a strong correlation (r = 0.83, *p* = 0.04). Linear regression (see Figure 2) showed a slope of 0.68 in WT, which was significantly different from zero (F_1,4_ = 29.19, *p* = 0.006). In KO, the slope was 0.12, which was significantly different from zero (F_1,4_ = 8.58, *p* = 0.04).

#### 3.1.7 Motor cortex

The motor cortex also revealed circadian rhythmicity with similar changes in receptor density in WT and M_4_KO animals (Figure 4). However, two-way ANOVA (interaction) (F_3,244_ = 0.8457, *p* = 0.47) did not find any differences between WT and M_4_KO animals (see also Table 2). There was no correlation between activity and the total number of mAChRs in WT (r = 0.77, *p* = 0.07), and similarly, in KO animals, there was also no correlation (r = 0.77, *p* = 0.07). Linear regression (see Figure 2) showed a slope of 0.58 in WT, which was not significantly different from zero (F_1,4_ = 5.7, *p* = 0.07). In KO, the slope was 0.12, which was not significantly different from zero (F_1,4_ = 5.88, *p* = 0.07).

#### 3.1.8 Relative M_4_ mAChR density

The changes in the relative density of M_4_ mAChRs were counted as the difference between the total mAChR density in WT and M_4_ KO animals.

**FIGURE 6:**
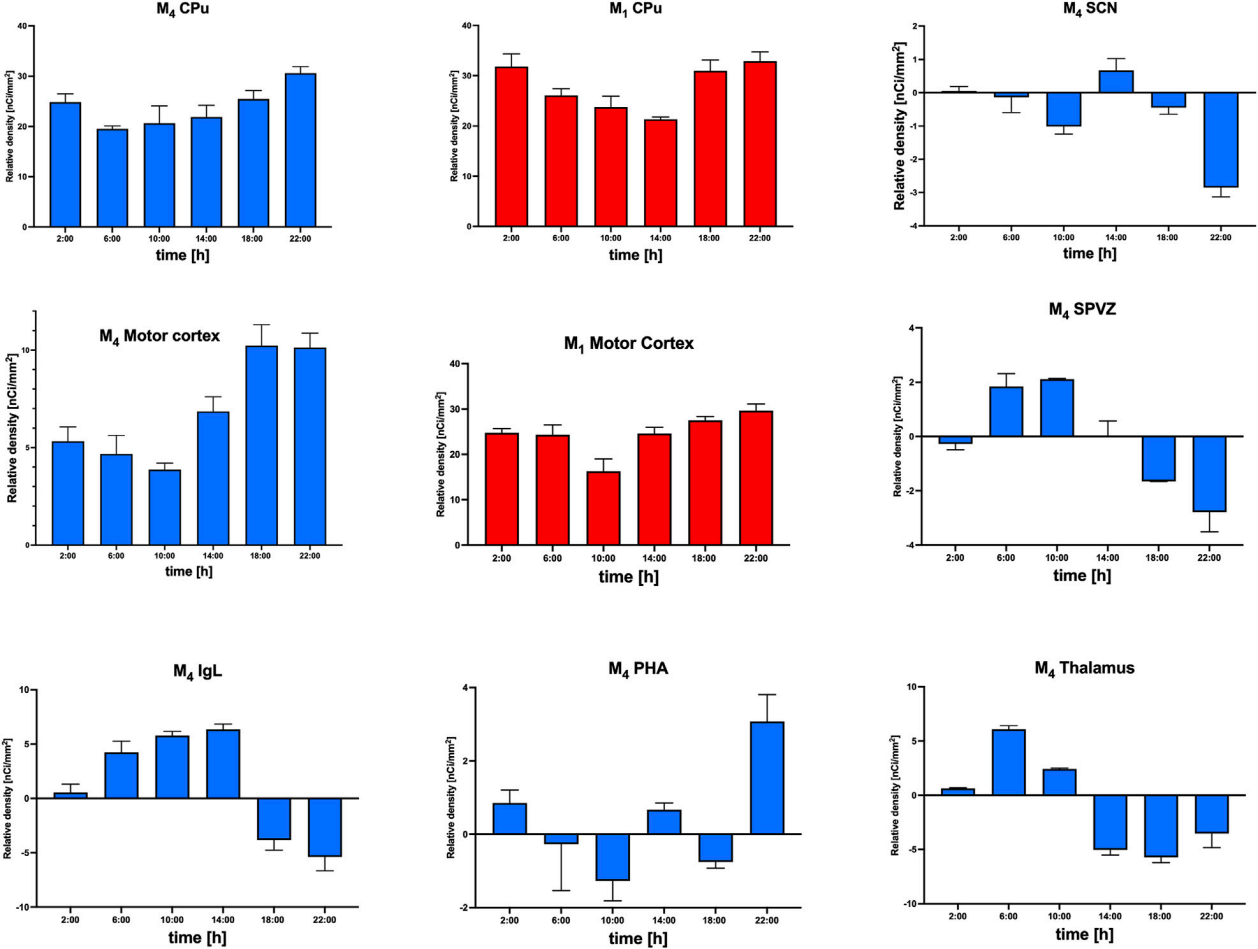
Relative density of M_1_ (red) and M_4_ (blue) mAChRs in the CPu and motor cortex and M_4_ mAChRs (blue) in SCN, SPVZ, IgL, PHA, and thalamus. Ordinate: relative density expressed as nCi/mm^2^; abscissa: the time [h]. See the sbrain area legend for specific data.

If the result of this subtraction is negative, then M_4_ mAChR deletion will lead to a compensatory increase in other mAChR subtype(s). These changes in specific brain areas are shown in Figure 6. As the receptor density in WT animals was obtained from different individuals than the values from KO animals, it is not possible to obtain individual points. Thus, the resulting value was the result of subtracting average values, and no individual points could be shown.

#### 3.1.9 Comparison of the total number of mAChRs and M_1_ mAChRs in caudate ncl.-putamen and ncl. accumbens

**FIGURE 7:**
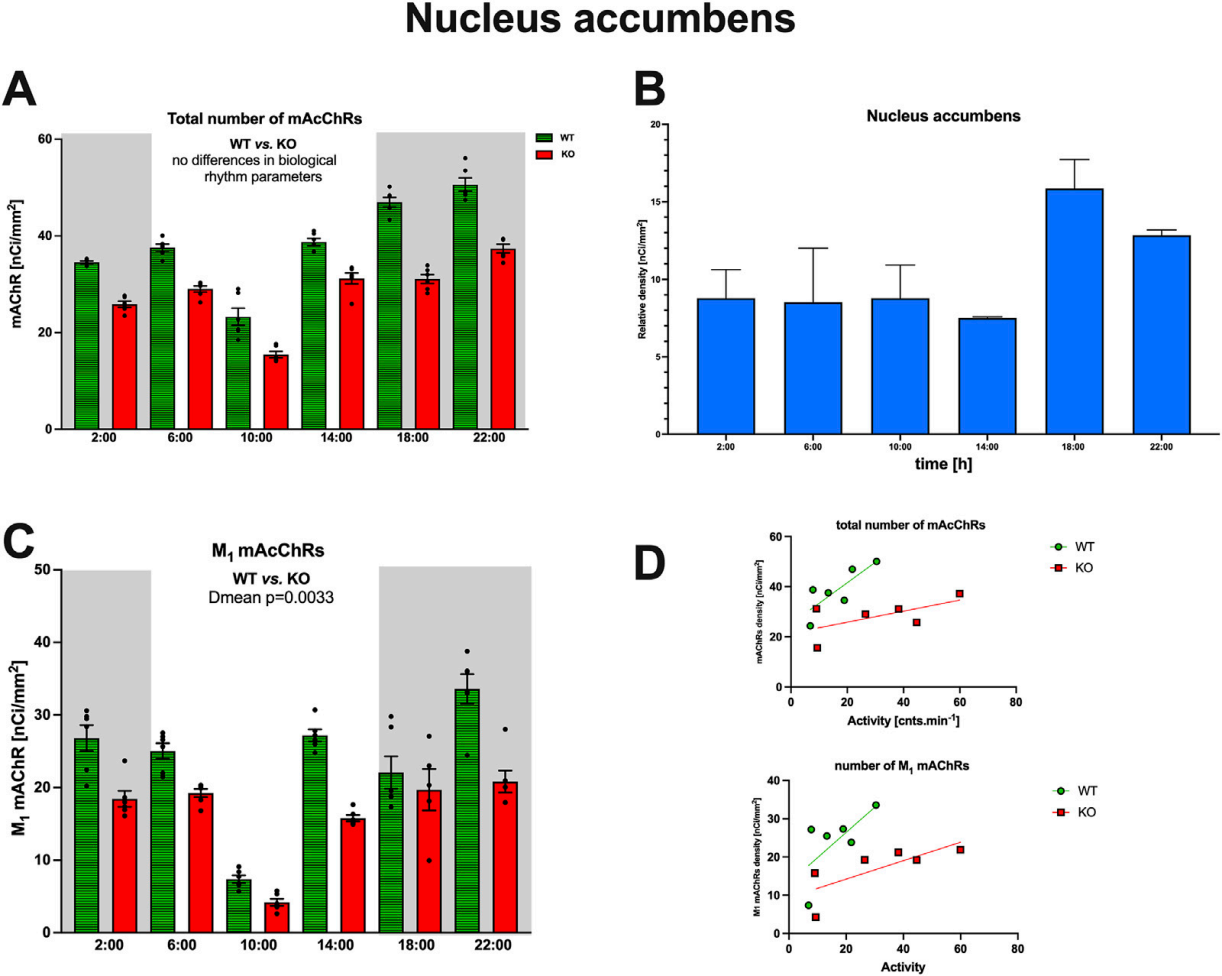
**(A)** Changes in the total number of mAChRs in the ncl. accumbens. Ordinate: relative density expressed as nCi/mm^2^; abscissa: time [h]. Grey rectangles represent the dark phase (lights off at 18:00 and lights on at 6:00). Data are expressed as the mean ± SEM with individual points. Two-way ANOVA: interaction between the genotype and biological rhythm parameters was not significant: F_3,825_ = 1.789, *p* = 0.15. The result of statistical analysis is given in the middle of the figure. Legend: WT, wild-type animals; KO, M_4_ mAChR KO animals. **(C)** Relative density of M_4_ mAChRs (blue). Ordinate: relative density expressed as nCi/mm^2^; abscissa: the time [h]. **(B)** Changes in the number of M_1_ mAChRs in the ncl. accumbens. Ordinate: relative density expressed as nCi/mm^2^; abscissa: time [h]. Grey rectangles represent the dark phase (lights off at 18:00 and lights on at 6:00). Data are expressed as the mean ± SEM with individual points. Two-way ANOVA: interaction between the genotype and biological rhythm parameters was significant: F_3,84_ = 13.12, *p* < 0.0001. The result of statistical analysis is given in the middle of the figure. Legend: WT, wild-type animals; KO, M_4_ mAChR KO animals. **(D)** The correlation between the total number of mAChRs or M_1_ mAChRs and locomotor activity. Top: the linear regression in WT and KO animals for the total number of mAChRs. Bottom: the linear regression in WT and KO animals for the number of M_1_ mAChRs. Ordinate: relative density expressed as nCi/mm^2^; abscissa: activity [cnts.min^-1^]. There was no significant correlation. Please note that in KO animals, there was higher locomotor activity. There was no correlation between activity and the total number of mAChRs in WT (r = 0.8, *p* = 0.054), and similarly, in KO animals, there was also no correlation (r = 0.61, *p* = 0.2). Linear regression (see Figure 7) showed a slope of 0.82 in WT, which was not significantly different from zero (F_1,4_ = 7.33, *p* = 0.054). In KO, the slope was 0.22, which was not significantly different from zero (F_1,4_ = 2.411, *p* = 0.19).

The density of the total number of mAChRs and M_1_ mAChRs was also measured in the ncl. accumbens, a structure not directly involved in locomotor activity but tightly connected to CPu. The total number of mAChRs was lower than in CPu; there was no difference in biological rhythm parameters, and there was no correlation with locomotor activity (see Figure 7). The total mAChR rhythmical activity in the ncl. accumbens is ultradian (both in WT and KO animals). The subtracted number of M_4_ mAChRs is lower than in CPu. In detail, two-way ANOVA (interaction): F_3,825_ = 1.789, *p* = 0.15 did not find any differences between WT and M_4_KO animals. There was no correlation between activity and the total number of mAChRs in WT (r = 0.8, *p* = 0.054), and similarly, in KO animals, there was also no correlation (r = 0.61, *p* = 0.2). Linear regression (see Figure 7) showed a slope of 0.82 in WT, which was not significantly different from zero (F_1,4_ = 7.33, *p* = 0.054). In KO, the slope was 0.22, which was not significantly different from zero (F_1,4_ = 2.411, *p* = 0.19). The number of M_1_ mAChRs was comparable with those in CPu; there was no difference in biological rhythm parameters, and there was no correlation with locomotor activity (see Figure 7). The rhythmical activity of M_1_ mAChRs in the ncl. accumbens is ultradian/circadian. In detail, two-way ANOVA (interaction): F_3,237_ = 5.32, *p* = 0.0015 revealed a difference between WT and M_4_KO animals in D_mean_ (*p* = 0.0033). There was no correlation between activity and the number of M_1_ mAChRs in WT (r = 0.67, *p* = 0.14), and similarly, in KO animals, there was also no correlation (r = 0.79, *p* = 0.06). Linear regression (see Figure 7) showed a slope of 0.66 in WT, which was not significantly different from zero (F_1,4_ = 3.29, *p* = 0.14). In KO, the slope was 0.26, which was not significantly different from zero (F_1,4_ = 6.58, *p* = 0.06).

### 3.2 M_1_ mAChRs number

The representative autoradiograms in WT and M_4_KO animals are shown in Supplementary Figure S3.

#### 3.2.1 Caudate nucleus-putamen

**FIGURE 8:**
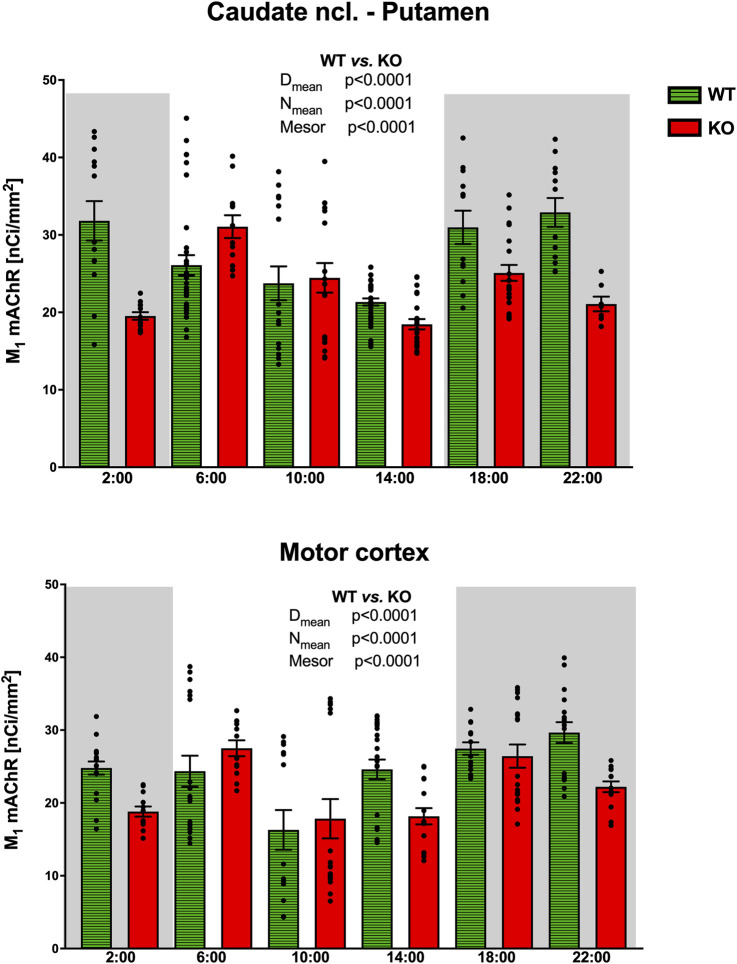
Changes in the number of M_1_ mAChRs in the striatum (top) and motor cortex (bottom). Ordinate: relative density expressed as nCi/mm^2^; abscissa: time [h]. Grey rectangles represent the dark phase (lights off at 18:00 and lights on at 6:00). Data are expressed as the mean ± SEM with individual points. Two-way ANOVA: interaction between the genotype and biological rhythm parameters was significant: F_3,84_ = 13.12, *p* < 0.0001 (striatum) and differences have been shown in the N_mean_ (*p* < 0.0001), D_mean_ (*p* < 0.0001), and mesor (*p* < 0.0001); F_3,112_ = 19.78, *p* < 0.0001 (motor cortex) and differences have been shown in the N_mean_ (*p* < 0.0001), D_mean_ (*p* < 0.0001), and mesor (*p* < 0.0001). The result of statistical analysis is given in the middle of the figure. Legend: WT, wild-type animals; KO, M_4_ mAChR KO animals.

**FIGURE 9:**
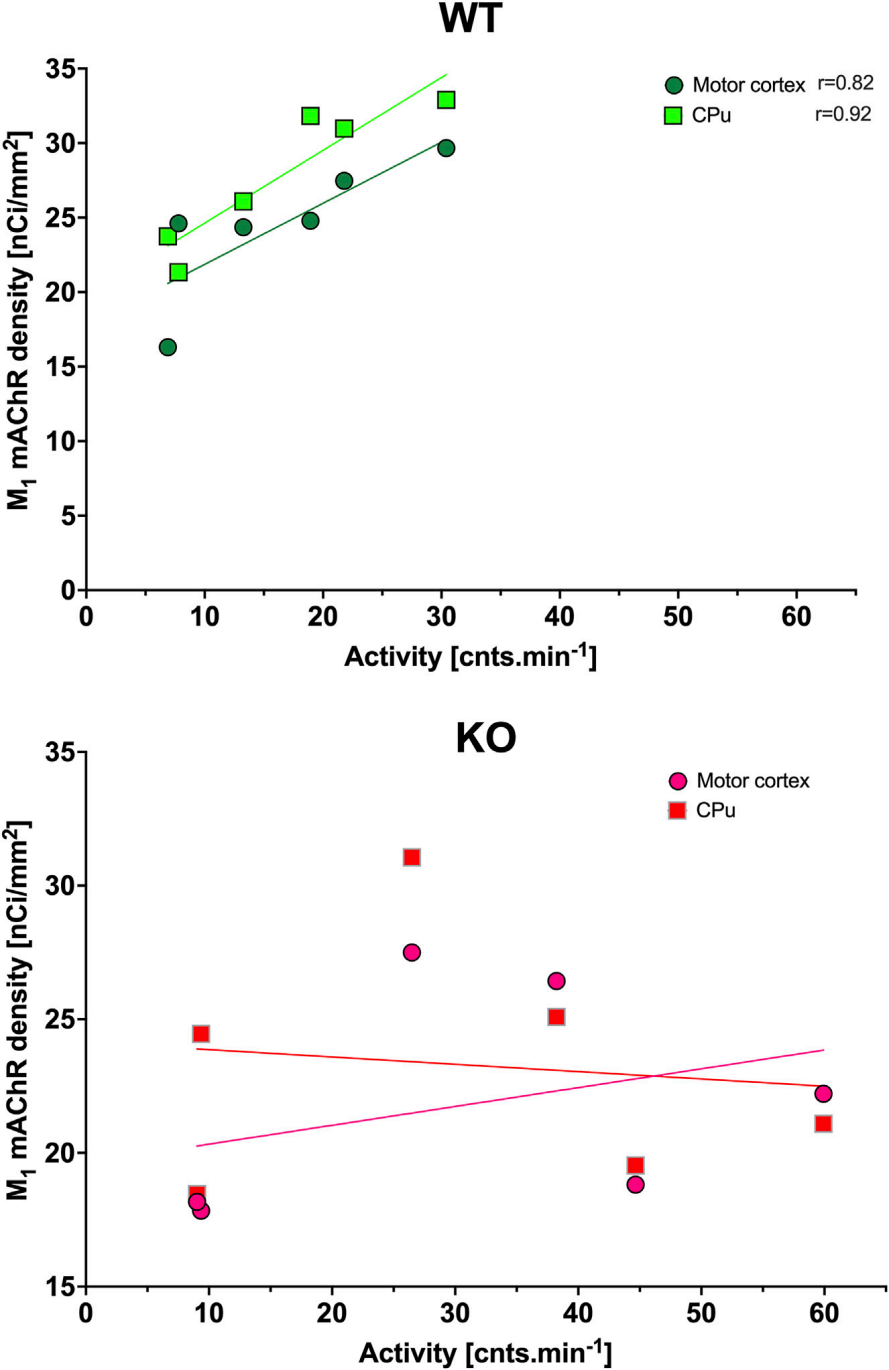
Correlation between the total number of M_1_ mAChRs and locomotor activity. Top: the linear regression in WT animals in the motor cortex and striatum. Bottom: the linear regression in KO animals in the motor cortex and striatum. Ordinate: relative density expressed as nCi/mm^2^; abscissa: activity [cnts.min^-1^]. Significant correlation coefficients are shown. Please note that in KO animals, there was higher locomotor activity.

In CPu (Figure 8), the rhythm in WT animals was circadian (with a peak at the end of the active phase, i.e., at 6:00), while M_4_KO animals revealed ultradian rhythm (with peaks at 6:00 and 18:00). Two-way ANOVA (F_3,84_ = 13.12, *p* < 0.0001) showed differences between D_mean_ (*p* < 0.0001), N_mean_ (*p* < 0.0001), and mesor (*p* < 0.0001). There was a strong correlation between locomotor activity and the number of M_1_ mAChRs in WT (r = 0.92, *p* = 0.008), while in KO animals, there was no correlation (r = −0.12, *p* = 0.82). Linear regression (see Figure 9) showed a slope of 0.49 in WT, which was significantly different from zero (F_1,4_ = 23.44, *p* = 0.008). In KO, the slope was −0.02, which was not significantly different from zero (F_1,4_ = 0.05, *p* = 0.82).

#### 3.2.2 Motor cortex

While WT animals had circadian rhythm in the motor cortex (Figure 8) with peaks at 22:00, M_4_KO animals had ultradian activity with peaks at 6:00 and 18:00 (thus the same as in the CPu). Two-way ANOVA (F_3,112_ = 19.78, *p* < 0.0001) showed differences between D_mean_ (*p* < 0.0001), N_mean_ (*p* < 0.0001), and mesor (*p* < 0.0001). There was a correlation between locomotor activity and the number of M_1_ mAChRs in WT (r = 0.82, *p* = 0.05), while in KO animals, there was no correlation (r = 0.33, *p* = 0.52). Linear regression (see Figure 9) showed a slope of 0.41 in WT, which was significantly different from zero (F_1,4_ = 7.92, *p* = 0.05). In KO, the slope was 0.07, which was not significantly different from zero (F_1,4_ = 0.5, *p* = 0.52).

#### 3.2.3 Density of M_1_ mAChRs in WT animals

The changes in the relative density of M_1_ mAChRs in the cortex and striatum, compared with the relative density of M_4_ mAChRs, are shown in Figure 6. The density of M_1_ mAChRs in M_4_ KO animals is taken from Figure 8 (WT animals). To unify this figure, individual values are not shown for M_1_ mAChRs.

### 3.3 Acetylcholinesterase activity

#### 3.3.1 Caudate nucleus-putamen

**FIGURE 10:**
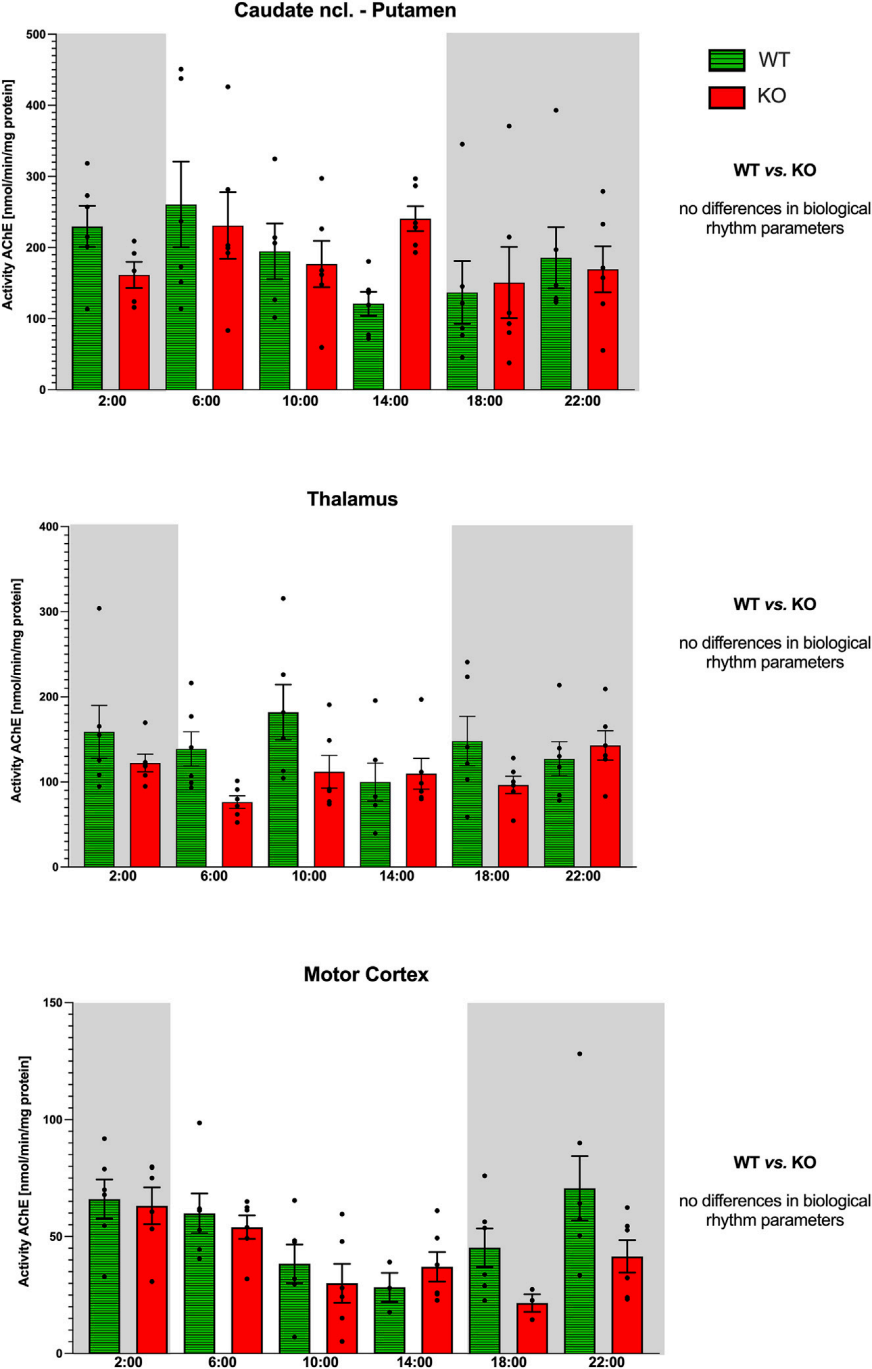
Activity of AChE (ordinate, expressed as nmol/min/mg protein) in the striatum (top), thalamus (middle), and motor cortex (bottom). Abscissa: time [h] and genotype. Grey rectangles represent the dark phase (lights off at 18:00 and lights on at 6:00). Data are expressed as the mean ± SEM. Two-way ANOVA: interaction between the genotype and biological rhythm parameters was not significant: F_3,40_ = 0.14, *p* = 0.94 (striatum), F_3,40_ = 1.21, *p* = 0.32 (thalamus), and F_3,40_ = 0.09, *p* = 0.96 (motor cortex). See legend for an explanation of symbols.

WT animals had a circadian rhythm with a peak at 6:00, and M_4_KO animals had ultradian activity (Figure 10) with peaks at 6:00 and 14:00. Two-way ANOVA (F_3,40_ = 0.13, *p* = 0.94) did not find any differences between WT and M_4_KO animals.

#### 3.3.2 Thalamus

Both WT and M_4_KO animals revealed ultradian activity (Figure 10). While in WT animals the peaks appeared at 2:00, 10:00, and 18:00, in M_4_KO animals, there were two peaks (at 10:00 and 22:00). Two-way ANOVA (F_3,40_ = 1.21, *p* = 0.32) showed no differences between WT and M_4_KO animals.

#### 3.3.3 Motor cortex

WT animals had a circadian rhythm with a peak at 22:00, and M_4_KO animals had ultradian activity (Figure 10) with peaks at 2:00 and 14:00. Two-way ANOVA (F_3,40_ = 0.09, *p* = 0.96) did not find any differences between WT and M_4_KO animals.

In all structures studied (caudate ncl-putamen, thalamus, and motor cortex), there were no correlations between AChE activity and locomotor activity in either WT or KO animals. In WT, the values were as follows: r = 0.04, *p* = 0.95; r = −0.11, *p* = 0.84; and r = 0.76, *p* = 0.08 in the caudate ncl-putamen, thalamus, and motor cortex, respectively. In KO animals, there were the following correlation coefficients: r = −0.59, *p* = 0.21; r = 0.72, *p* = 0.11; and r = 0.31, *p* = 0.54 in the caudate ncl-putamen, thalamus, and motor cortex, respectively. Similarly, linear regression showed non-significant values.

### 3.4 Butyrylcholinesterase activity

#### 3.4.1 Caudate nucleus-putamen

**FIGURE 11:**
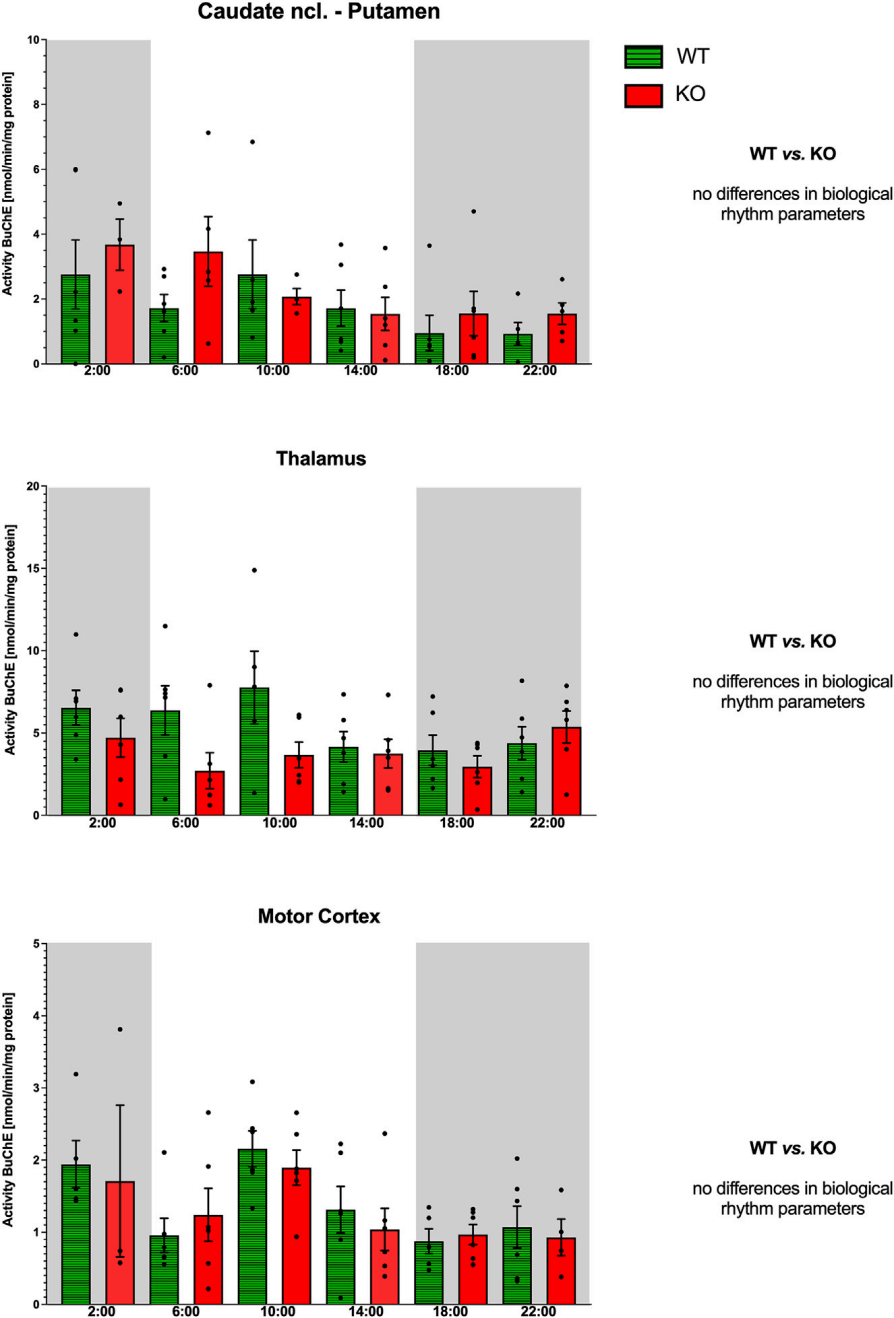
Activity of BuChE (ordinate, expressed as nmol/min/mg protein) in the striatum (top), thalamus (middle), and motor cortex (bottom). Abscissa: time [h] and genotype. Grey rectangles represent the dark phase (lights off at 18:00 and lights on at 6:00). Data are expressed as the mean ± SEM. Two-way ANOVA: interaction between the genotype and biological rhythm parameters was not significant: F_3,38_ = 1.21, *p* = 0.32 (striatum), F_3,40_ = 0.28, *p* = 0.84 (thalamus), and F_3,40_ = 0.05, *p* = 0.99 (motor cortex). See legend for an explanation of symbols.

WT animals had an ultradian rhythm with peaks at 2:00 and 10:00, and M_4_KO animals had a circadian rhythm with a peak at 2:00. Two-way ANOVA (F_3,38_ = 1.24, *p* = 0.32) was not significant (Figure 11).

#### 3.4.2 Thalamus

Both WT and M_4_KO animals had ultradian rhythms (Figure 11) with peaks at 2:00 and 10:00 (WT) and 14:00 and 22:00 (M_4_KO). Two-way ANOVA (F_3,40_ = 0.28, *p* = 0.84) did not find any differences between WT and M_4_KO animals.

#### 3.4.3 Motor cortex

Both WT and M_4_KO animals had ultradian rhythms (Figure 11) with peaks at 2:00 and 10:00. Two-way ANOVA (F_3,40_ = 0.05, *p* = 0.99) did not find any differences between WT and M_4_KO animals.

In all structures studied (caudate ncl-putamen, thalamus, and motor cortex), there were no correlations between BuChE activity and locomotor activity in either WT or KO animals. In WT, the values were as follows: r = −0.48, *p* = 0.19; r = −0.61, *p* = 0.34; and r = −0.46, *p* = 0.36 in the CPu, thalamus, and motor cortex, respectively. In KO animals, there were the following correlation coefficients: r = 0.08, *p* = 0.88; r = 0.59, *p* = 0.22; and r = −0.35, *p* = 0.50 in the CPu, thalamus, and motor cortex, respectively. Similarly, linear regression showed non-significant values.

## 4 Discussion

In this study, we show, to the best of our knowledge, the presence of biological rhythms of mAChRs and cholinesterases in brain areas specific to the regulation of locomotor activity in a cyclic manner. To our knowledge, the biological rhythms of mAChRs and cholinesterases have not been described in the SCN, IgL, SPVZ, PHA, and thalamus. We believe that this is also the first report on the role of different brain areas in the mAChRs-directed biological rhythm of locomotor activity. Generally, it is also necessary to mention that we described the biological rhythm using time points (tissue was collected every 4 h). This is a compromise between animal consumption and the need to obtain as many points as possible. As mentioned, Cosinor analysis is unsuitable as it could produce a bias. When we have tentatively performed MATLAB cubic spline interpolation, the results were mostly consistent with the conclusions we report in our manuscript. Therefore, we limited our conclusions to stating whether the rhythm is clearly circadian in nature or whether its frequency is higher, and then we referred to this finding as an ultradian rhythm.

We found that mAChRs peaks in control animals are mostly present in the dark (active) phases (i.e., in the SCN, PHA, thalamus, CPu, and motor cortex) or shortly after the end of the dark phase (IgL). The only structure having a peak in the light (inactive) phase was SPVZ. In addition to that, M_1_ mAChRs had a peak in the dark phase, both in the CPu and motor cortex. This agrees with studies and reviews on mAChRs in the striatum or forebrain (Wirz-Justice et al., 1981; Kafka et al., 1983; Wirz-Justice, 1987) in rats, although these authors also identified an additional peak in the light phase. Similarly, the circadian rhythmicity of mAChRs was detected in the hypothalamus in the study by Por and Bondy (1981) in rats, although they did not find circadian rhythmicity in the cerebral cortex, striatum, or cerebellum. Biological rhythms in mAChRs (with a peak in the subjective night, i.e., in the active period) were found in the olfactory bulb, parietal cortex, and caudate ncl-putamen (Kafka et al., 1986). This study also found no rhythms in the frontal or occipital cortex, nucleus accumbens, hippocampus, thalamus–septum, pons–medulla, or cerebellum. When we compared the total mAChR density in the CPu and ncl. accumbens, we found lower densities in the ncl. accumbens with ultradian rhythm. The densities of M_1_ mAChRs were comparable in the caudate ncl-putamen and ncl. accumbens, the biological rhythm was ultradian in WT and circadian in KO. No correlation was found between locomotor activity and the total/M_1_ mAChRs number. The biological rhythm in mAChR density was not found in the rat forebrain and brainstem (Mash et al., 1985). Completely different results were reported by Hut and Van der Zee (2011): while most brain areas showed mAChRs peak during the inactive phase (Marquez et al., 1990), they observed the peak of mAChRs in rats during the non-active period. In Syrian hamsters (Bina et al., 1998), no detectable rhythm was found in the anterior hypothalamus (including the SCN). The differences could probably be attributed to the early studies employing membrane binding, while we have used autoradiography, which is generally more suitable for central nervous system studies. Thus, to the best of our knowledge, the presence of an mAChR biological rhythm in the SCN is a novel finding. Earlier (Valuskova et al., 2018b), we characterized mAChR density in the SCN as low and found no differences between WT and KO animals. This very low density could also result in the finding of rhythm absence in this brain structure, as described earlier (Bina et al., 1998).

The biological rhythm detection of M_1_ mAChRs also represents new findings. However, it has been suggested that M_1_ mAChRs play a role in the biological rhythm of spontaneous nervous activity in the SCN (Gillette et al., 2001) in the night phase of rats. McN-A-343 caused a phase advance shift (Basu et al., 2016). In this study, the authors declare that McN-A-343 is an M_1_/M_4_ agonist, but it tends to act as a positive allosteric ligand and exhibits an order of effect: M_4_>M_3_>M_2_>M_1_>M_5_ (Myslivecek, 2022). At the concentration used (79 mM, i.e., 10^−4.1^ mol/L), it can activate all mAChRs, but it is reasonable to assume that M_4_ and M_3_ are fully activated, while other subtypes are activated by approximately 50%. Similarly, McN-A-343 caused an SCN phase shift in the subjective night that was blocked by pirenzepine, suggesting the involvement of M_1_ mAChRs (Liu and Gillette, 1996). However, McN-A-343 is not an M_1_ mAChR-selective ligand and neither is pirenzepine at the concentrations used in this study. Here, we demonstrated the circadian rhythm of M_1_ mAChRs in the CPu and motor cortex that was changed to ultradian in KO animals. Importantly, we have shown that in both the CPu and motor cortex, there was a strong correlation between locomotor activity and the number of M_1_ mAChRs in WT but not in KO animals. These results show the interplay between M_1_ and M_4_ mAChRs in the CPu and motor cortex. Deletion of M_4_ mAChRs disrupts the influence of M_1_ mAChRs on locomotor activity biological rhythm, suggesting the major role of M_4_ mAChRs in the locomotor activity biological rhythm. The role of M_4_ mAChR is, as described earlier (Valuskova et al., 2018b), inhibitory. In mAChR KO animals, it has been shown (Oki et al., 2005) that the deletion of one mAChR subtype is compensated for by changes in other subtypes. This could also be a possibility of no correlation between locomotor activity and the number of M_1_ mAChRs in KO animals. Taken together, we can assume the role of M_4_ mAChRs in muscarinic receptor-directed locomotor activity biological rhythm as major, with the role of M_1_ mAChRs being secondary.

The representation of relative density (Figure 6) showed parallel changes in M_1_ and M_4_ mAChRs in the motor cortex. However, in the CPu, the changes in M_1_ and M_4_ mAChR density did not occur in parallel. In other structures (SCN, SPVZ, PHA, and thalamus), the subtraction revealed negative values for M_4_ mAChRs, suggesting the involvement of another muscarinic subtype (compensatory increase) in the biological rhythm regulation when M_4_ mAChRs are deleted. As verified by VU6013720 (a truly selective M_4_ mAChR antagonist), binding in M_4_ KO animals represents the binding to other muscarinic receptors than M_4_ mAChRs. Thus, when deletion of M_4_ mAChRs (there is no binding to M_4_ mAChRs in M_4_ KO animals, as verified by VU6013720) led to a decrease in binding, there must be other subtype(s) that are compensatorily increased in M_4_ KO, leading to a negative result of subtraction: mAChRs density_WT_ − mAChRs density_KO_.

The biological rhythm of AChE is in partial agreement with the study of plasma AChE activity in female rats (Alves-Amaral et al., 2010), where the peak was also evident at 8:00 p.m. (12 h/12 h regime, lights on at 6:00 a.m.) and another peak at 6:00 a.m. We have found the peak at 22:00 in the motor cortex and 6:00 in the CPu. It should be stressed that this is a comparison of plasma (Alves-Amaral et al., 2010) and central nervous system activities (our data), and thus, this partial agreement is quite interesting. The review (Hut and Van der Zee, 2011) concluded that acetylcholinesterase activity had its peak in the inactive phase. On the other hand, differences in amplitude and phase relationships among the markers have been reported between brain regions and strains. We found a peak in AChE activity in the inactive phase in the thalamus, while in the CPu and motor cortex, AChE activity peaked in the active phase. The thalamus revealed an ultradian rhythm, while the motor cortex and CPu revealed a circadian rhythm. Knocking out M_4_ mAChRs shifted the rhythm to ultradian. In the mouse brain stem reticular formation, there was a significant ultradian rhythm of AChE activity (Lewandowski, 1988). Our analysis did not show any correlation between AChE activity and locomotor activity. We can thus conclude that AChE is not essential for locomotor activity biological rhythms in the specific brain areas studied.

The biological rhythm detection of BuChE activity in the central nervous system is a new finding. The function of BuChE is still a matter of debate (Zhe Ying et al., 2020). BuChE has lower acetylcholine catalytic efficiency. Initial studies on butyrylcholinesterase showed that the inhibition of the enzyme led to an increase in brain acetylcholine levels. Later, BuChE has been shown to change the amount of amyloid-beta (for review, see Zhe Ying et al. (2020)). It has also been shown that BuChE plays a role in the stress reaction in the CNS (Valuskova et al., 2017).

Previously, BuChE activity was studied in rat plasma (Alves-Amaral et al., 2010), which found an ultradian rhythm with peaks at 8:00 a.m. and 8:00 p.m. Our results in the CNS also showed an ultradian rhythm of BuChE activities in the CPu and motor cortex, with peaks at 2:00 a.m. and 10:00 a.m., while in the thalamus, there was a circadian rhythm with a peak at 10:00 a.m. Knocking out M_4_ mAChRs changed the rhythm (see Figure 11). Our analysis did not show any correlation between BuChE activity and locomotor activity. We can thus conclude that BuChE is not essential for locomotor activity biological rhythm in specific brain areas studied.

Our results are also in agreement with the finding that SCN muscarinic receptors are involved in circadian clock regulation (Yang et al., 2010). These authors used an electrophysiological experiment with McN-A-343 (declared as an M_1_/M_4_ agonist (see also above) and blocked by the M_4_ antagonist: MT3 toxin). As mentioned above, carbachol caused a phase shift in locomotor activity. In addition to this, carbachol could cause a non-photic phase shift when injected into the IgL (Cain et al., 2007). LY2033298 (Gannon and Millan, 2012), a positive allosteric modulator at muscarinic M_4_ receptors, enhances inhibition when applied together with intraperitoneally applied oxotremorine (nonselective muscarinic agonist) on light-induced phase shifts in hamster circadian activity rhythms. However, it is necessary to stress that LY2033298 also binds with similar affinity to M_2_ muscarinic receptors (Myslivecek, 2022). Our results show that the deletion of M_4_ mAChRs leads to a strong correlation between the number of mAChRs and locomotor activity, suggesting a suppressive role of M_4_ mAChRs in the regulation of locomotor activity biological rhythm in the SCN.

**FIGURE 12:**
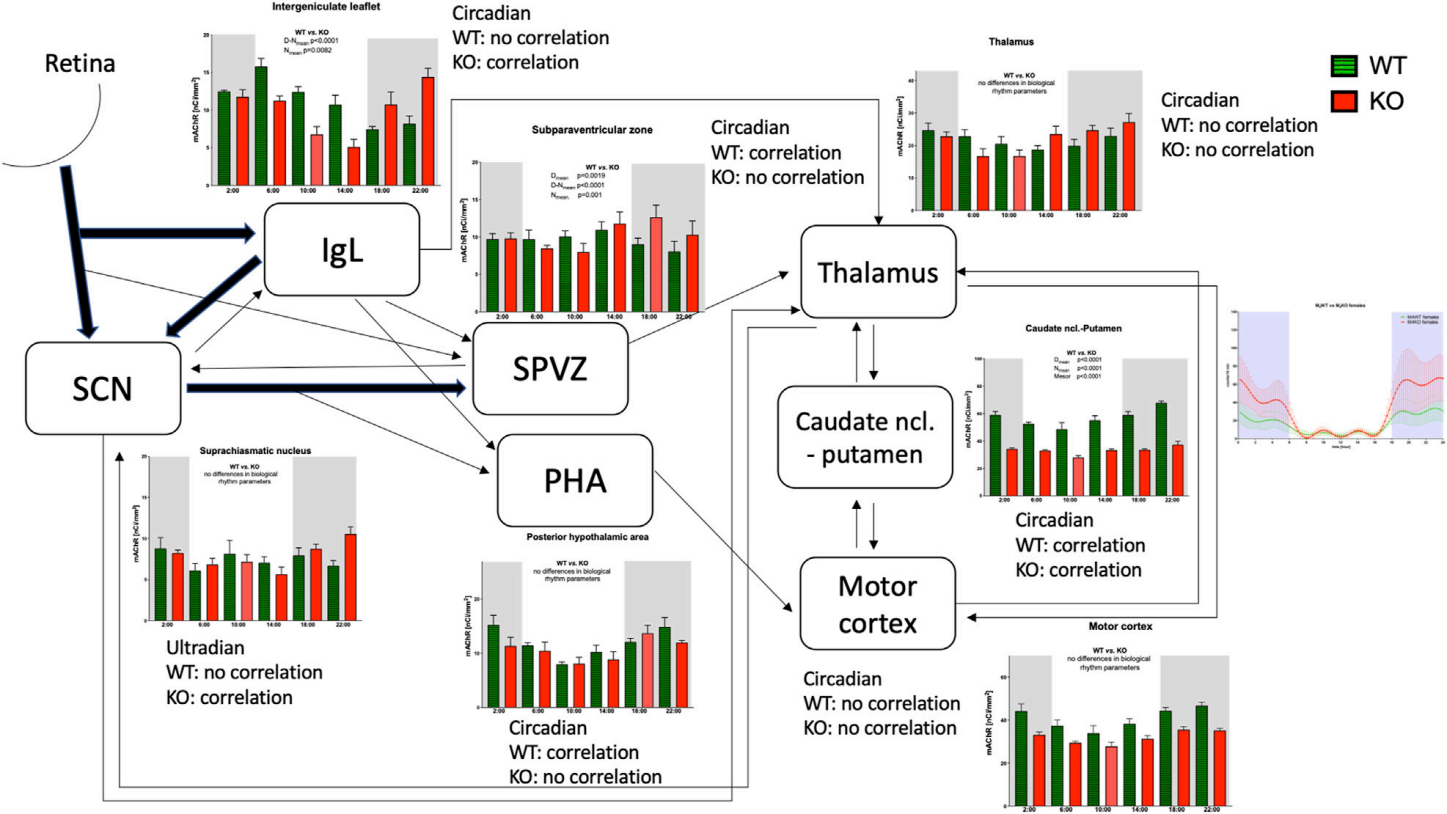
Schematic diagram showing the connections between brain areas with the biological rhythm of the total number of muscarinic receptors. The line thickness shows the strength of the connection. The correlation between receptor density and locomotor activity is shown by word correlation/no correlation. The locomotor output was taken from our previous work (Valuskova et al., 2018b).

Considering the projections between structures involved in locomotor activity biological rhythm (see also Table 1), it is possible to summarize (see Figure 12) that the SCN, under the influence of photosensitive cells in the retina, produces ultradian rhythms of the total number of mAChRs present in this brain area. This rhythm is changed to circadian in the IgL, which is influenced by the collateral fibers from the retinohypothalamic tract and receives additional input from the SCN (see Introduction for more details). Vice versa, the IgL sends significant efferents to the SCN. In both the SCN and IgL, deleting M_4_ mAChRs leads to a strong positive correlation between the number of mAChRs and locomotor activity, suggesting a suppressive role of M_4_ mAChRs in regulating locomotor activity biological rhythm.

In other hypothalamic structures, the SPVZ and PHA, the biological rhythm of the total number of mAChRs remains circadian. Contrary to IgL, in these structures, the biological rhythm of mAChRs revealed a strong correlation with locomotor activity in WT (it was a negative correlation in SPVZ and a positive correlation in PHA), and this correlation disappeared in KO animals. The SPVZ receives inputs from the SCN, IgL, and collateral fibers from the retinohypothalamic tract. The PHA receives collaterals from the connection between the SCN and SPVZ; the IgL and SPVZ send efferent fibers to the thalamus and the PHA to the motor cortex.

Both in the thalamus and motor cortex, the circadian rhythm in mAChRs can be found. In addition to that, the SCN reciprocally communicates with the thalamus. Other connections exist between the CPu, thalamus, and motor cortex. The CPu seems to be the key structure in motor activity biological rhythm as there is an evident decrease in the total number of mAChRs showing circadian rhythm in M_4_KO animals, which almost copies the biological rhythm in WT animals. Moreover, this is the only structure in which a correlation was found the correlation between the biological rhythm of total mAChRs and the activity biological rhythm in WT and KO. In WT, there was also a correlation between the biological rhythm of M_1_ mAChRs number and the biological rhythm of locomotor activity.

Other molecules involved in cholinergic transmission (AChE and BuChE) play minor, auxiliary, or no roles in the determination of locomotor activity biological rhythm.

Generally, the changes in the biological rhythm parameters in specific structures are lower than twofold (see Table 2). However, one should consider that the final output—locomotor activity—reveals biological rhythm parameters (Valuskova et al., 2018b) that are nearly doubled: the mesor increased from 16.06 to 30.7 (i.e., 1.91 times), the night mean increased from 25.58 to 54.26 (i.e., 2.12 times), and the night–day mean increased from 18.64 to 47.14 (i.e., 2.52 times). Thus, it is not surprising that changes in the receptor number are slightly lower than the resulting change in locomotor activity. We can thus view the changes in locomotor activity as a result of additive effects of receptor changes.

In conclusion, we revealed the sequence of changes in brain areas involved in mAChR regulation of locomotor activity.

We describe here the sequence of changes in specific brain areas leading to increased locomotor activity in M_4_ KO female mice during their dark phase. Initially, the ultradian rhythm of muscarinic receptors in the suprachiasmatic nucleus is changed to circadian in the intergeniculate leaflet, subparaventricular zone, and posterior hypothalamic area, revealing different phase shifts in these brain structures. Subsequently, the CPu, thalamus, and motor cortex, with a major role of the CPu, transform this rhythm to increased locomotor activity in mice. The main role in the muscarinic receptor-directed biological rhythm of activity is played by striatal M_4_ muscarinic receptors, with a contribution of M_1_ muscarinic receptors. Cholinesterases play a minor or no role in this regulation. These are new findings describing the role of muscarinic receptors in the regulation of locomotor activity.

## Data Availability

The raw data supporting the conclusions of this article will be made available by the authors, without undue reservation.

## Abbreviations

AchE, acetylcholinesterase; BuChE, butyrylcholinesterase; CPu, caudate ncl-putamen; D_mean_, day mean; IgL, intergeniculate leaflet; M_4_KO, M_4_ knockout animals; mAChRs, muscarinic receptors; mesor, midline value of biological rhythm; N–D_mean_, difference between night and day mean; N_mean_, night mean; PHA, posterior hypothalamic area; SCN, suprachiasmatic nucleus; SPVZ, subparaventricular zone; WT, wild-type animals.
