# Supplementary figures and images for: Striatal M4 muscarinic receptors determine the biological rhythm of activity, with a supportive role of M1 muscarinic receptors
Source: Front Pharmacol. 2025 Dec 1;16:1691118. doi: 10.3389/fphar.2025.1691118 (PMC12702858; doi:10.3389/fphar.2025.1691118)

Figure 2

Autoradiography QNB

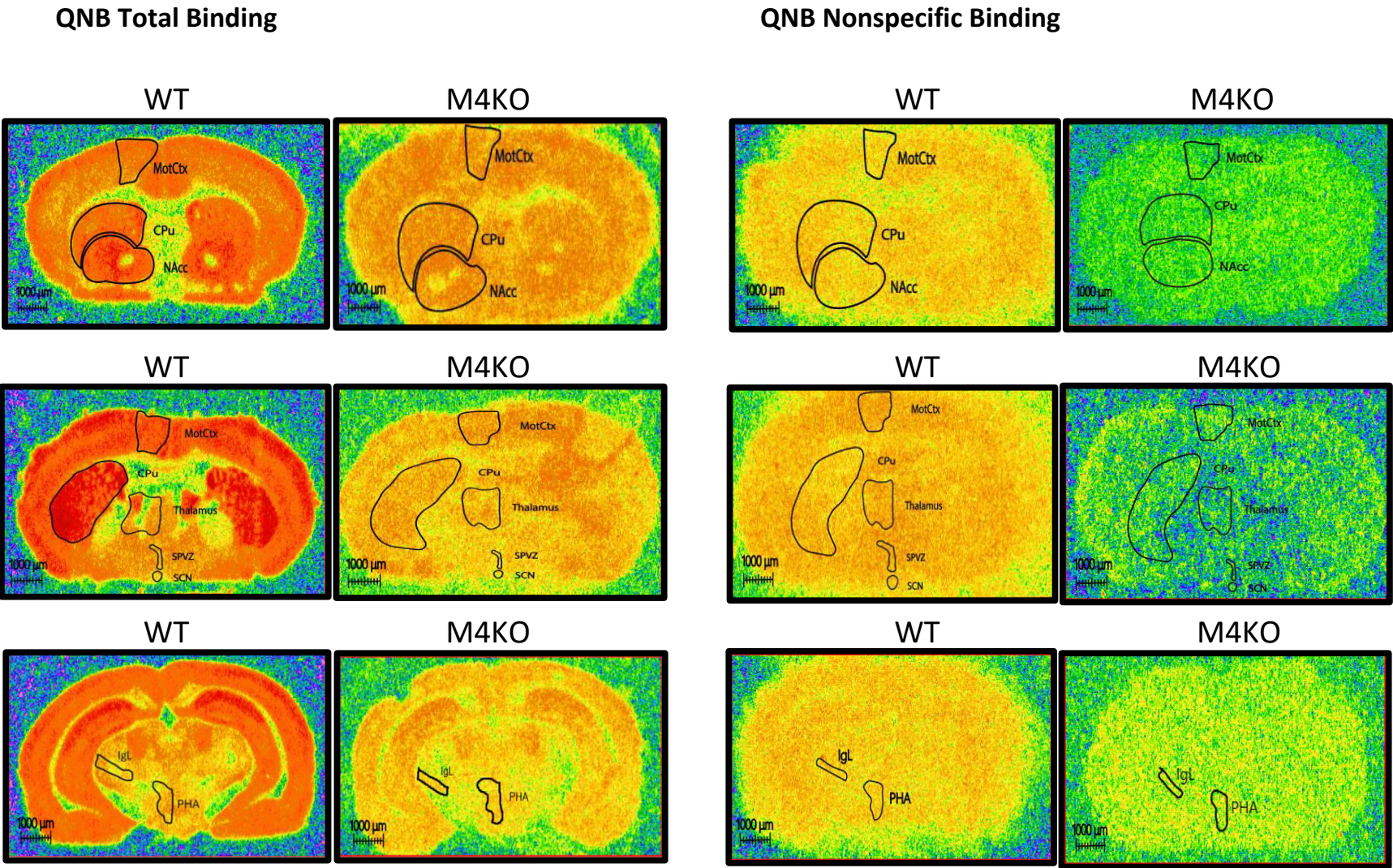

Supplement: Supplementary file 1 [file DataSheet2.pdf]

Figure 1

## Nissl Staining

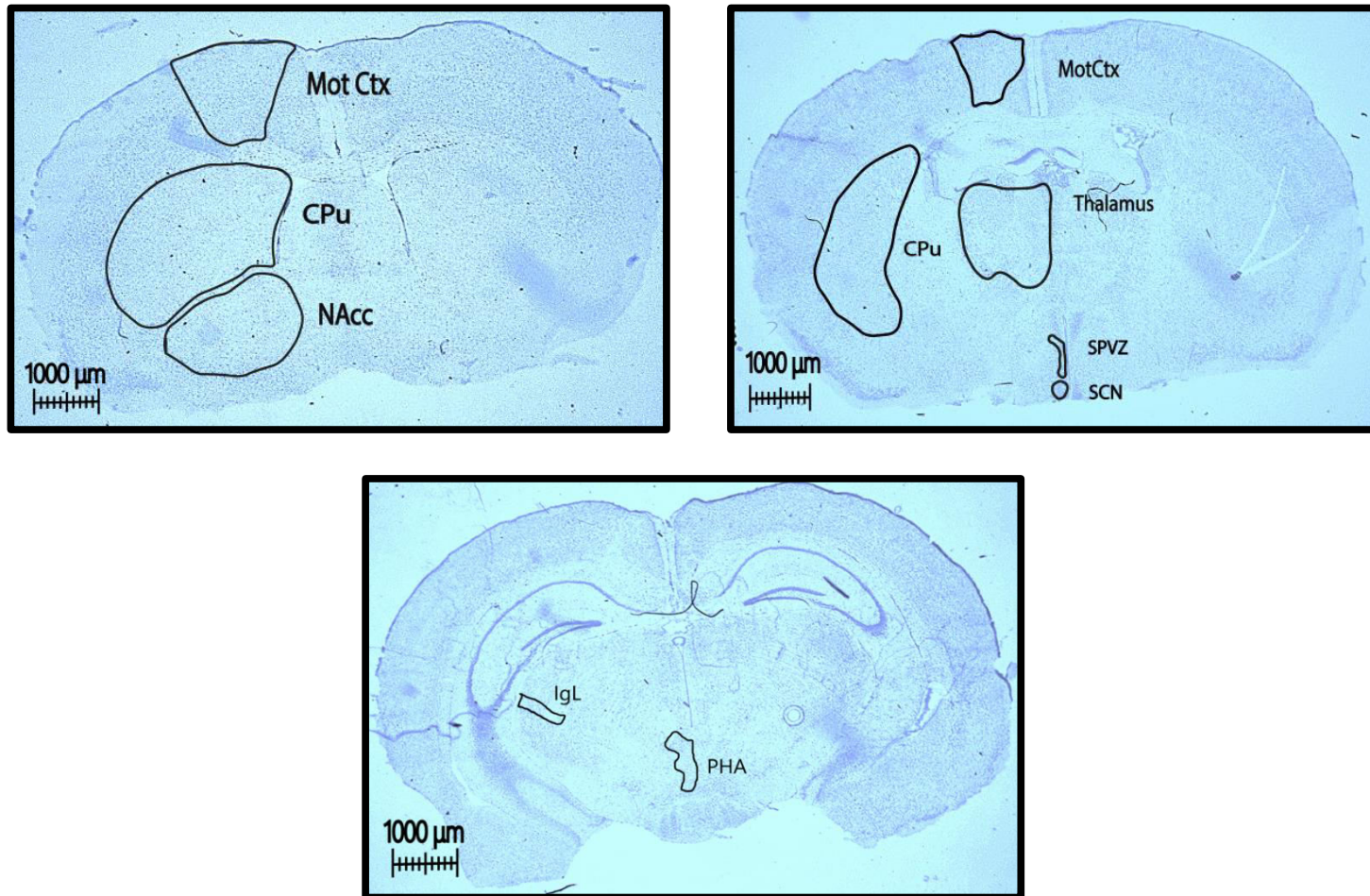

Supplement: Supplementary file 3 [file DataSheet1.pdf]
