# Supplementary material for: Striatal M4 muscarinic receptors determine the biological rhythm of activity, with a supportive role of M1 muscarinic receptors
Source: Front Pharmacol. 2025 Dec 1;16:1691118. doi: 10.3389/fphar.2025.1691118 (PMC12702858; doi:10.3389/fphar.2025.1691118)

Figure 3

Autoradiography Pirenzepine

Pirenzepine Total Binding **Pirenzepine Nonspecific Binding**

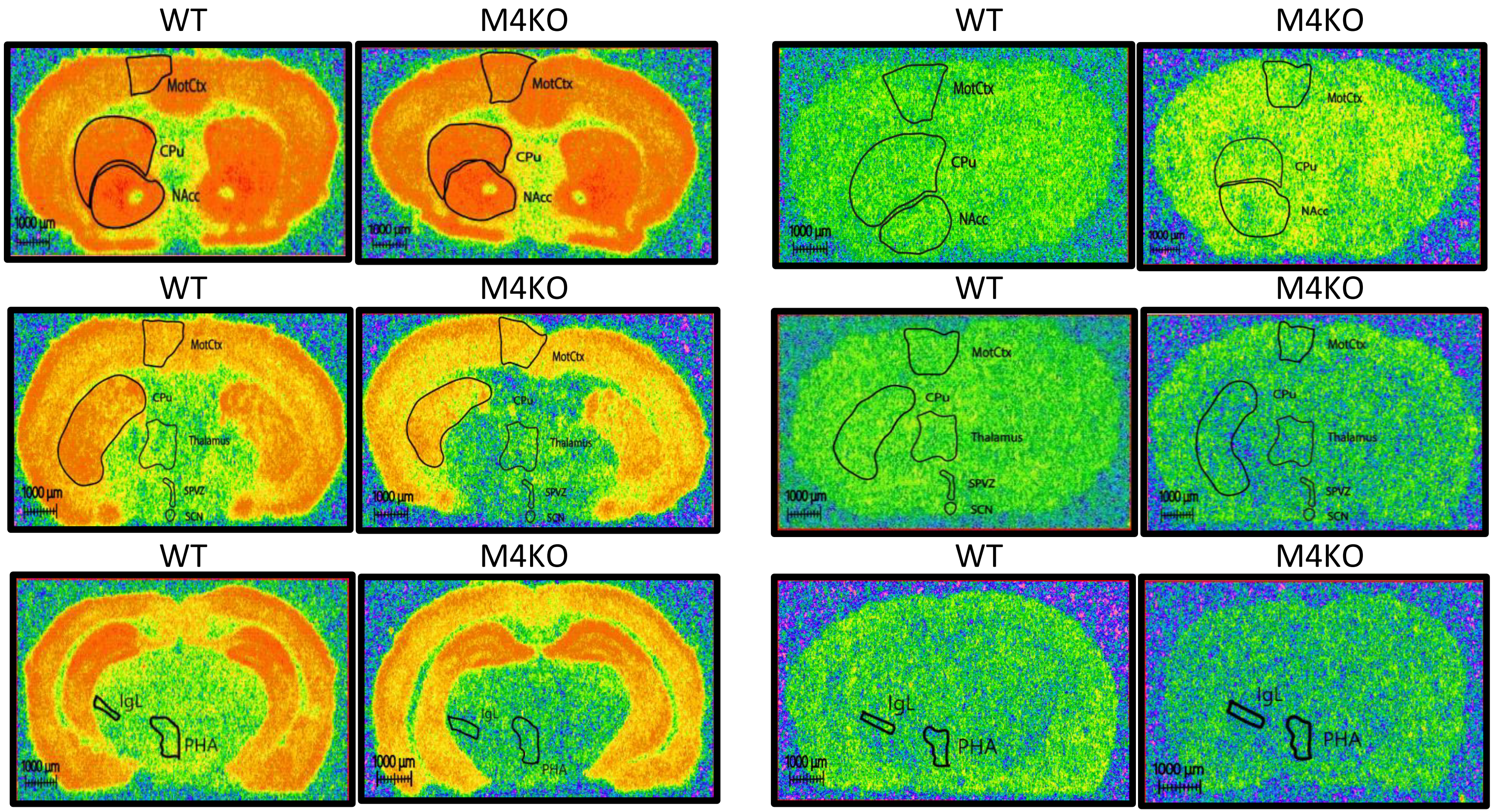

Supplement: Supplementary file 2 [file DataSheet3.pdf]
